# Tree species mixing can increase stand productivity, density and growth efficiency and attenuate the trade-off between density and growth throughout the whole rotation

**DOI:** 10.1093/aob/mcab077

**Published:** 2021-06-22

**Authors:** H Pretzsch, G Schütze

**Affiliations:** Chair for Forest Growth and Yield Science, TUM School of Life Sciences, Weihenstephan, Technical University of Munich, Hans-Carl-von-Carlowitz-Platz 2, 85354 Freising, Germany

**Keywords:** Tree species mixing, stand productivity, stand density, growth efficiency, competition reduction, facilitation, overyielding, overdensity, trade-off between stand productivity and tree growth

## Abstract

**Background and Aims:**

Many recent studies emphasize that mixed species is a promising silvicultural option for sustainable ecosystem management under uncertain and risky future environmental conditions. However, compared with monocultures, knowledge of mixed stands is still rather fragmentary. This comprehensive study analysed the most common Central European tree species combinations to determine the extent to which mono-layered species mixing (1) can increase stand productivity and stem diameter growth, (2) increase stand density or growth efficiency, and (3) reduce competition and attenuate the relationship between stand density and stem diameter growth compared with mono-specific stands.

**Methods:**

The study was based on 63 long-term experimental plots in Germany with repeated spatially explicit stand inventories. They covered mono-specific and mixed species stands of Norway spruce (*Picea abies*), silver fir (*Abies alba*), Scots pine (*Pinus sylvestris*), European beech (*Fagus sylvatica*), sessile oak (*Quercus petraea*), European ash (*Fraxinus excelsior*) and sycamore maple (*Acer pseudoplatanus*). Based on spatially explicit measurement, we quantified for each tree the intra- or inter-specific neighbourhood, local stand density and growth. We applied mixed models to analyse how inter-specific neighbourhoods modify stand productivity, stand density, growth efficiency, individual tree growth and the trade-off between individual tree growth and stand productivity.

**Key Results:**

We found stand productivity gains of 7–53 % of mixed versus mono-specific stands continuing over the entire rotation. All mixtures achieved a 3–36 % higher leaf area index until advanced stand age. Stem diameter growth increased by up to 31 % in mixed stands. The growth efficiency of the leaf area was up to 31 % higher, except in mixtures of sessile oak and European beech. The trade-off between stem diameter growth and stand productivity was attenuated by the mixture.

**Conclusions:**

The increased productivity was mainly based on a density increase in the case of Norway spruce/silver fir/European beech and sessile oak/European beech and it was based on a more efficient resource use given the same stand density in the case of Scots pine/European beech and European ash/sycamore maple. In the other species assemblages the increased productivity was based on a combination of density and efficiency increase. We hypothesize that the density effect may be site-invariant and mainly depends on the structural species complementarity. The efficiency increase of growth may depend on the growth-limiting factor that is remedied by mixture and thus be co-determined by the site conditions. For forest management, the results indicate increased stand and tree size growth by species mixing. For the common mixtures examined in this study the results show that thinning for the acceleration of stem growth requires less density reduction and causes less stand growth losses than in monocultures. We discuss the consequences of our findings for silvicultural prescriptions for mixed-species stands.

## INTRODUCTION

Many recent studies emphasize that mixed species is a promising silvicultural option for sustainable ecosystem management under uncertain and risky future environmental conditions.

Recent studies suggest that mixed-species stands can overyield mono-specific stands (Mason and Connolly, 2014; Liang *et al.*, 2016; Jactel *et al.*, 2018) because they can be denser (Pretzsch, 2014; Jucker *et al.*, 2015; Steckel *et al.*, 2019) and more efficient in resource use (Khanna, 1997; Forrester, 2014). The superiority of mixed-species stands can even increase under drought (Lebourgeois *et al.*, 2013; Pretzsch *et al.*, 2013; Neuner *et al.*, 2015; Dănescu *et al.*, 2018), insect attacks (Jactel and Brockerhoff, 2007; Jactel *et al.*, 2021) and other types of abiotic and biotic disturbances (Knoke *et al.*, 2008; Griess *et al.*, 2012). For successful management of mixed-species stands (Pretzsch and del Río, 2020), especially for species selection and stand density regulation, it is important to understand the overyielding of different species assemblages, i.e. whether overyielding is mainly an effect of increased density or higher efficiency of resource use and how any mixing effects change with progressing stand development. Better insight into the structure and functioning of mixed-species stands is crucial for the exploitation of overyielding to improve wood production, carbon uptake, and storage. However, compared with monocultures, the knowledge about mixed stands summarized in this introduction is still rather fragmentary.

Several recent studies have found overyielding in middle-aged stands. Jactel *et al.* (2018), for instance, reported overyielding of 15 % in a meta-analysis. The overyielding reported by Pretzsch *et al*. (2010, 2015, 2020), Thurm and Pretzsch (2016), Steckel *et al.* (2019) and Ruiz-Peinado *et al.* (2021) ranged between 2 and 59 % in terms of stand volume or mass growth. The course of individual tree growth has been well analysed for mono-specific stands (Kozlowski, 1962; Zeide, 1993) and also for rich structured selection forests where trees can remain for a long period in the understorey (Magin, 1959; Mitscherlich, 1970; Hilmers *et al.*, 2019). Recent studies showed that tree mixing of species often (Brooks *et al.*, 2002; Pretzsch *et al.*, 2013) but not always (Grossiord, 2020; Gillerot *et al.*, 2020) facilitates tree growth in drought years, and that the mixing effects depend on the species identity and combination (Pardos *et al.*, 2021). However, how mixing modifies tree growth in the long term has been hardly addressed yet (Pretzsch and Schütze, 2009; Thurm *et al.*, 2017; Pretzsch *et al.*, 2021); this is probably due to the rarity of long-term observations.

So far, mixing effects on productivity have mainly been analysed in young or middle-aged stands. Young stands are often not yet closed, are far from maximum stand density, and have only a short time to acclimate to the intra- or inter-specific conditions (Bauhus *et al.*, 2000; Amoroso and Turnblom, 2006; Forrester *et al.*, 2006; Vanclay *et al.*, 2013). With progressing stand development the acclimation of trees to their neighbourhood may proceed differently in mixed compared with mono-specific stands. Therefore, comparisons between young mixed stands and respective monocultures (commonly used as reference) can hardly be transferred to older or mature forest stands (Nichols *et al.*, 2006). Most of the studies addressed in the previous paragraph and meta-analyses (Piotto, 2008; Pretzsch *et al.*, 2017; Jactel *et al.*, 2018) were based on medium-aged forest stands, which are often fully stocked, and represent the most productive phase of rotation. As long-term mixing experiments are rare, little is known about the mixing effects until advanced stand ages. Dieler *et al.* (2017) and Zeller and Pretzsch (2019) showed that in the advanced development stage, structural diversity may increase productivity (older stands may tend to open canopy space, caused by human and natural disturbances) and that when stands are mixed and structured, productivity losses may be better buffered and compensated than in mono-layered monocultures.

Several studies provide evidence that tree species mixing can increase stand density in terms of stand density index (Pretzsch and Biber, 2016; Williams *et al.*, 2017), crown coverage (Pretzsch, 2014) and leaf area index (LAI) (Peng *et al.*, 2017). Such an increase in packing density may result from the complementary use of space and resources above or below the ground (Forrester, 2014). Supposing there is mainly a density effect (competition reduction) of mixing on growth, the benefit of mixture could be exploited by keeping stands at a higher stand density level, whereas more widely planted or strongly thinned mixed stands would not generate more yield.

An increase in growth efficiency (growth per leaf area or per crown projection area), in contrast, means that at lower densities the inter-specific neighbourhood may increase the efficiency of the crown, similar to a fertilization effect (Khanna, 1997). In this case, benefits may emerge independently of stand density, but also under wide spacing and strong thinning. Thinning may cancel the density effect, but not the efficiency increase (Forrester *et al.*, 2013; Brunner and Forrester, 2020). Forrester *et al.* (2013) showed that the efficiency effect may be amplified by density, i.e. complementarity effects may become stronger under high density, or complementarity enables higher densities, so that both are positive and can reinforce each other.

For mono-specific stands, it is well known that stand density lowering reduces stand growth and increases individual tree diameter growth (del Río *et al.*, 2017), resulting in a well-known trade-off between stand productivity and tree size growth (Zeide, 2001; Mäkinen and Isomäki, 2004*a*, *b*; Pretzsch, 2020). In mixed stands, stem diameter growth and stand growth may be increased by competition reduction and facilitation (Kelty, 1992; Forrester, 2014). In mixed stands, larger stem diameters may be achieved even under higher stand densities than in mono-specific stands. Thus, a given target stem size may be achieved with higher density and less loss of stand productivity due to density reduction. The dilemma of the forest manager between maintaining stand density and growth at a maximum and providing tall high-quality stems by stand density reduction may be attenuated by tree species mixture.

The objective of this study was to comprehensively quantify common tree species combinations in Central Europe in terms of their tree and stand growth compared with mono-specific stands. Our study covered mono-specific and mixed species stands of Norway spruce (*Picea abies*), silver fir (*Abies alba*), Scots pine (*Pinus sylvestris*), European beech (*Fagus sylvatica*), sessile oak (*Quercus petraea*), European ash (*Fraxinus excelsior*), and sycamore maple (*Acer pseudoplatanus*).

We analysed 63 plots covering the species combinations of (1) Norway spruce/European beech, (2) Norway spruce/silver fir/European beech, (3) Norway spruce/Scots pine, (4) Scots pine/European beech, (5) sessile oak/European beech and (6) European ash/sycamore maple. The plots provided new insights as they represented, for each species combination, age series from young to mature stands at the same sites. All plots were fully stocked with the species growing in both inter- and intra-specific neighbourhoods. The plots represented medium- and high-quality site conditions; the range of site conditions was not wide enough for thoroughly exploring the dependency of the mixing effects on site quality. The measurements included tree coordinates, crown width and length, and up to five repeated surveys of stem diameter, and height of all trees. Tree heights and height to the crown base were measured at each survey but only of sample trees; crown sizes were measured only at one or two surveys but of all trees. The plots were used for analysis at the stand and tree levels and for testing the following hypotheses about mixed stands compared with mono-specific stands:

H I: Tree species mixing can increase stand productivity and stem diameter growth throughout the entire rotation.
H II: The mixing effects are based on both the increased stand density and growth efficiency.
H III: Tree species mixing can reduce competition and attenuate the relationship between stand density and stem diameter growth compared with mono-specific stands.

## MATERIALS AND METHODS

### Study plots

**Fig. 1.**
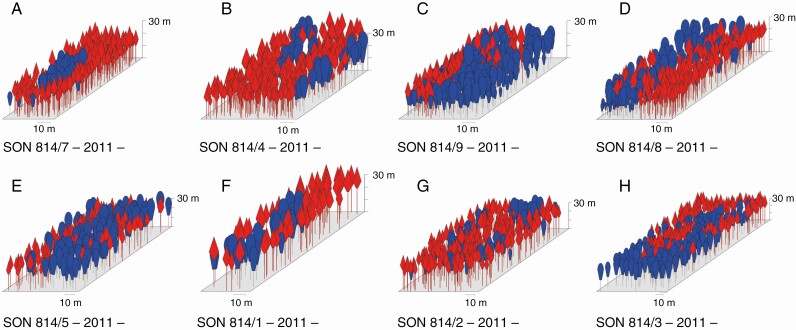
Age series SON 814 with eight plots near Schongau/Bavaria as an example of the setup of the 11 age series included in this study. (A–H) Plots 7, 4, 9, 8, 5, 1, 2 and 3 ranked by age, increasing from 62 to 127 years in the case of Norway spruce and 92 to 142 years in the case of European beech (state of the last survey in autumn 2011). Crown sizes of Norway spruce and European beech are plotted in red and blue, respectively. The sizes of plots 7, 4, 9, 8, 5, 1, 2 and 3 were 0.20, 0.41, 0.39, 0.39, 0.56, 0.26, 0.63 and 1.00 ha, respectively.

The study was based on 11 age series (see example in Fig. 1) with 63 long-term plots in Germany with repeated spatially explicit stand inventories. They were established in 18- to 238-year-old stands and covered the main tree species in Central Europe in intra- and inter-specific neighbourhoods throughout the whole rotation. The plots represented the most common medium- and high-quality site conditions (Table 1), were fully stocked, and the mixing patterns ranged from individual trees to cluster mixtures. Most of the stands were planted and even-aged; occasionally natural regeneration may have complemented them. On some of the plots moderate thinning from above was applied in the second half of the 20- to 30-year survey period.

**Table 1.** Location, climate characteristics and site conditions of the eight long-term experiments included in this study

Age series	Name	Species combination	Longitude	Latitude	Elevation a.s.l.	Annual precipitation	Mean temperature (°C)	Soil type	Substrate	Geology	Ecoregion (see footnote)
Unit			°	°	m	mm year^−1^	°C				
FRE 813	Freising	N. sp., E. be.	11·66	48·42	515	814	7·7	Parabrown soil	Loam	Tertiary sand	12.8
SON 814	Schongau	N. sp., E. be.	10·77	47·87	790	1114	6·8	Brown soil	Loam	Günz-Mindel lower moraine	14.4.1
NOR 811	Nordhalben	N. sp., E. be.	11·59	50·31	590	850	5·5	Brown soil	Stony loam	Clay shale	8.1
KEH 804	Kelheim	S. oak, E. be.	11·76	48·93	455	721	7·5	Brown soil	Silt loam	Tertiary sediments	6.2
ROT 801	Rothenbuch	S. oak, E. be.	9·44	49·95	375	960	7·0	Brown soil	Silt loam	Lower sandstone	2.2.1
SWE 803	Schweinfurt	S. oak, E. be.	10·30	50·13	340	660	8·0	Brown soil	Silt loam	Lower Trias	4.1
KRE 824	Kreuth	N. sp., s. fir, E. be.	11·69	47·63	1200	1800	4·5	Brown soil	Loam	Main dolomite	15.5
GEI 832	Geisenfeld	S. pi., E. be.	11·22	48·57	430	725	7·6	Brown soil	loamy sand	tertiary sand	12.8
AMB 833	Amberg	S. pi., E. be.	11·83	49·35	480	650	7·5	Brown soil	Sandy loam	Chalkstone	6.5
NEU 841	Neuburg	S. pi., N. sp.	11·22	48·56	430	725	7·6	Brown soil	Loamy sand	Tertiary sand	12.8
ARN 851	Arnstein	E. ash, syc. map.	9·94	49·99	260	670	8·0	Parabrown soil	Silt loam	Shell limestone	4.2

The ecoregion numbers indicate the following units (translation to English in brackets): 12.8 Oberbayerisches Tertiärhügelland (Upper Bavarian tertiary hills), 14.4.1 Westliche kalkalpine Jungmoräne (Western limestone young moraine region), 8.1 Frankenwald (Franconian Forest), 6.2 Südlicher Oberpfälzer Jura (Southern Upper Palatinate Jurassic region), 2.2.1 Hochspessart (Upper Spessart region), 4.1 Nördliche Fränkische Platte (Northern Franconian plateau region), 15.5 Mittlere Bayerische Kalkalpen (Middle Bavarian limestone Alps), 6.5 Oberpfälzer Jurarand (Upper Palatinate jurassic borderline region), 4.2 Südliche Fränkische Platte (Southern Franconian plateau region) (according to Arbeitskreis Standortskartierung (1985) Forstliche Wuchsgebiete und Wuchsbezirke in der Bundesrepublik Deutschland).

N. sp., Norway spruce; E. be. European beech; s. oak, sessile oak; s. fir, silver fir; S. pi., Scots pine; E. ash, European ash; syc. map., sycamore maple.

The age series were established in the 1990s for *ad ho*c data acquisition for parameterization of an individual tree simulator for mono-specific and mixed species stands in South Germany (Pretzsch *et al.*, 2002). The plots within each age series were established on similar sites and close to each other. Since their establishment and first survey, chronosequences were remeasured up to five times, so that the original chronosequences became real-time series of long-term surveys. For example, the survey of a 20-year-old stand (first survey carried out 25 years ago) was repeated four times so that the original 20-year-old stand resulted in a 45-year-old one and overlapped with the survey data of the original 40-year-old stand. In this way, we covered, for all considered mixtures, an age span of a whole rotation or more.

The pseudo 3-D visualization of the age series SON 814 in Fig. 1 was based on the first inventory and tree coordinate measurements in 1991; the repeated measurements (survey 2011) of the stem diameters, tree heights and crown sizes are explained in detail in the next section. Supplementary Data Fig. S1 shows as an example the crown maps of SON 814/1–8, where the plots of the age series cover individual trees and group-mixture Norway spruce and European beech as well as mono-specific parts. For the sake of simplicity, we visualized the crown size as concentric circles calculated as the quadratic mean of the eight crown radius measurements recorded during the course of the repeated surveys.

The plot size increased from the young to the old stands (see 10-m scale at the bottom of each of the crown maps) in order to cover representative sections of the representative stand development phases. The range of plot sizes varied according to stand ages between 0.2 and 1.0 ha and was similar between age series. The plots within the age series covered stand ages from 17 to 238 years. The experiment KRE824 represents a large plot that comprises several age phases in close vicinity to each other.

### Dendrometric measurements

**Fig. 2.**
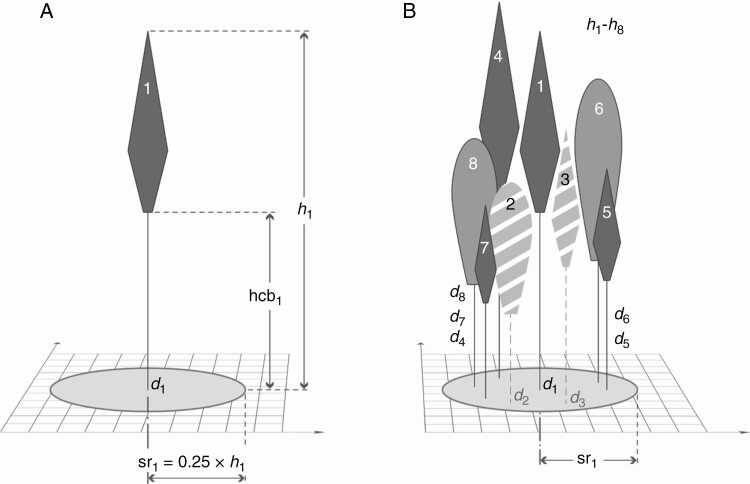
(A) Measurements at tree level. (B) Setup for the evaluation of the local neighbourhood of each tree. For variable explanation see footnote Table 2. The central tree in the circle has number 1 and the breast height diameter and tree height *d*_1_ and *h*_1_. The search radius around the central tree has the radius sr_1_. In this example there are the neighbouring trees numbered 2–8, with diameters *d*_2_–*d*_8_, and heights *h*_1_–*h*_8_ within the search radius. Trees 2 and 3 (hatched) represent removal trees.

Table 2 summarizes the abbreviations and explanations of the main measurement variables and metrics and the objective variables used in this study. From each tree that was higher than 1·30 m, we recorded the species identity, measured the *x*- and *y*-coordinates of the tree positions at the first survey, and all stem diameters at breast height in each of the up to five surveys (Fig. 2A, Table 3). Tree height (*h*) and height to crown base (hcb) of a subset of trees were measured in each survey. For this purpose, we sampled ~30 trees per species on each plot on each age series; at the plot level the sample trees were selected uniformly over the whole diameter range. In the course of the successive surveys we preferably used the same sample trees for the measurement of height and height to crown base. However, we replaced them by neighbours of similar stem diameter if they had been removed. Crown radii in the eight cardinal directions were measured only in one or two surveys, but for all trees. All these introduced tree characteristics were also measured for the ingrowth since the first survey. In addition, we inventoried the natural regeneration since the first survey; as the latter information was not used for this study we refrain from reporting details about the natural regeneration.

**Table 2.** Overview of main measurements variables and metrics used in this study

Variable and metric names	Abbreviation	Explanation and indication
(1) Tree-level variables		
Stem diameter	*d*	Indication of tree present size
Tree height	*h*	Determination of radius for competition analysis
Height to crown base, to lowest branch	hcb	Indication of bole length, used for visualization
Crown radius	cr	$\overset{-}{cr}=\sqrt{(r_{1}^{2}+r_{2}^{2}+\ldots +r_{8}^{2})/8}$ for visualization
Crown length	cl	cl=h−hcb used for visualization
Tree leaf area	la	Estimated depending on stem diameter
Above-ground tree mass	ma	Estimated depending on stem diameter
Search radius for neighbourhood analysis	sr	$sr_{1}=0.25\times h_{1}$ for analysis
Annual stem diameter increment	id	Periodical diameter increment/period length
Local competition index	SDIci	Local SDI in circle calculated without centre tree
Local mixing proportion	mixport	Calculated using equivalence factors
(2) Variables related to sample circle		
Stand density index on circle	SDIc	SDI standardized by equivalence coefficients
Mass growth on circle	IMc	Scaled up by stem diameter, tree mass >7 cm
Quadratic mean tree diameter on circle	dqc	Species overarching dq
Leaf area index at circle level	LAIc	LAI standardized by equivalence coefficients
Categorical variable indicating mono-specific versus mixed on circle	*m*	*m *= 0, i.e. mixing proportion <10 % *m *= 1, i.e. mixing proportion ≥10 %
(3) Stand-level variables		
Quadratic mean stem diameter	dq	Calculated species-overarching
Standing stem volume	*V*	Merchantable volume 7 cm at the smaller end
Leaf area index	LAI	Upscaled depending on *d* by allometric functions
Carbon stock at the stand level	*C*	Above-ground biomass × 0·5
Stand stem volume growth	IV	Periodical mean annual stem volume growth

**Table 3.** Overview of the age 11 series with 63 plots included in this study. The number of measurements refers to the number of measured tree attributes, such as stem diameters, coordinates crown and characteristics at the first survey; remeasurements are only counted once. For explanation of species names see footnote of Table 1

Age series	Name	Species combination	Site index^a^ ho age 100	Age from to^b^	Number of plots	Total plot area	First survey	Last survey	Number of surveys	Number of trees measured for *d* including repeated	Number of trees measured for *h* including repeated	Number of trees measured, *x*, *y* coordinates	Number of trees measured, crowns including repeated
Unit			m	years		ha	m			Number	Number	Number	Number
FRE 813	Freising	N. sp., E. be.	35·1	37–168	6	2·87	1994	2012	4	7939	2498	2725	2020
SON 814	Schongau	N. sp., E. be.	37·4	50–142	8	3·87	1991	2011	5	14106	4204	3619	3538
NOR 811	Nordhalben	N. sp., E. be.	33·3	36–126	5	2·19	1997	2008	2	3474	1494	1736	999
KEH 804	Kelheim	S. oak, E. be.	32·7	17–165	7	3·18	1996	2015	4	14587	2743	4129	1426
ROT 801	Rothenbuch	S. oak, E. be.	25·6	32–238	6	3·35	1994	2009	3	11282	1270	3911	1389
SWE 803	Schweinfurt	S. oak, E. be.	27·3	21–186	6	2·78	1995	2005	2	6874	1813	4000	1339
KRE 824	Kreuth	N. sp., s. fir, E. be.	20·5	50–218	1	0·87	1994	2012	4	2503	526	724	759
GEI 832	Geisenfeld	S. pi., E. be.	32·0	18–214	6	2·07	1996	2010	3	7823	1837	2775	1029
AMB 833	Amberg	S. pi., E. be.	31·5	26–136	5	0·69	1991	2016	5	5142	1942	1869	979
NEU 841	Neuburg	S. pi., N. sp.	32·8	22–118	6	2·46	1997	2014	3	13433	4002	5135	1406
ARN 851	Arnstein	syc. map., E. ash	35·0	20–94	7	1·93	1998	2014	3	5557	2864	5135	1241

^a^ho is related to the leading tree species mentioned in the first place in column 3.

^b^Relates to the first survey of the youngest plot and the last survey of the oldest plot.

The stand age was read off from the historical documentation of stand establishment; if this was not available we derived the tree age by tree-ring counting on increment cores sampled at the foot of the trunks. Stand ages were assumed to be identical with mean tree age in cases of natural regenerated stands. In planted stands, stand age was assumed to be mean tree age minus 3 years to take into account the usual age of plants coming from the nursery.

### Rationale of the descriptive evaluation

Testing the hypotheses H I to H III required dendrometric characteristics at the tree level, information about the neighbourhood of the trees, and growth and yield characteristics of the stands (see variables in Table 2). For completion of the tree-level data we used auxiliary relationships, e.g. for deriving the tree height, stem mass and leaf area of every tree. For analysing the competition and neighbourhood we constructed a sample circle around each tree and analysed, among others, the local stand density and the species composition within this circle. Stand-level growth and yield characteristics were derived on both the sample circle level and the whole plot level. In the following sections we describe the auxiliary relationships and methods which we applied for the derivation of the various characteristics.

### Auxiliary relationships

By modelling the relationship between height, stem diameter and age (see section Results. Overview of tree and stand characteristics), we calculated the height of each tree. To estimate the individual tree height (*h*) depending on the stem diameter (*d*) and tree age (age) we parameterized the model below for each species in each of the 11 age series:


$$\begin{array}{ll} \ln\left(h\right)= & \;a_{0}+a_{1}\times \ln\left(d\right)+a_{2}\times \ln\left(age\right)\\ & +a_{3}\times \ln\left(d\right)\times \ln\left(age\right) \end{array}$$ (1)

All regression coefficients were significant, at least at the level of *P* < 0.05; the *R*^2^ values ranged between 0.85 and 0.98. For the model parameters, see Supplementary Data Table S1.

To estimate the above-ground stem biomass (ma) and leaf area (la) we used the species-specific functions of Forrester *et al.* (2017):


$$\begin{array}{l} \ln\left(ma\right)=a_{0}+a_{1}\times \ln\left(d\right) \end{array}$$ (2)

$$\begin{array}{l} \ln\left(la\right)=a_{0}+a_{1}\times \ln\left(d\right) \end{array}$$ (3)

Equations (2) and (3) result in the de-logarithmic models $ma=exp(a_{0}+a_{1}\times \ln\left(d\right))\times CF_{m}$ and $la=exp(a_{0}+a_{1}\times \ln\left(d\right))\times CF_{la}$. Factors CF were applied in order to correct for the bias that results from back-transforming ln-transformed predictions of the biomass (ma) and leaf area (la) (Snowdon, 1991). For the model parameters, see Supplementary Data Tables S2 and S3. For species with minor portions for which species-specific functions were not available, we used the generalized functions for conifer and broad-leaved species, respectively (see also Forrester *et al.*, 2017). Above-ground tree carbon mass resulted from multiplication of above-ground tree dry biomass by 0·50 (Knigge and Schulz, 1966; Körner, 2002; Lamlom and Savidge, 2003).

### Analysing tree competition and neighbourhood

For analysing the effects of intra- and inter-specific neighbourhoods on stem diameter growth of each tree we constructed a circle with radius sr_1_ = 0·25 × *h*_1_ around its stand point, where $h_{1}$ is the height of the central tree (Fig. 2B). Within the constructed circles, there were, on average, eight or nine trees and at least five or six neighbours with strong impact on the growth of the central tree (Prodan, 1968*a*, b). We fixed the search radius to a quarter of the height of the respective central tree as other search radii resulted in lower correlations between growth of the central tree and the respective competition index. By choosing the search radius depending on tree height we took into consideration that in even-aged stands the influence zone around a tree increases with progressing size development (Pretzsch, 2009, pp. 295–296). All trees within the constructed circle were used to quantify local competition, mixing status, local density, leaf area and productivity.

To quantify the competitive status of each individual tree we calculated the local stand density index (SDIci). This calculation was based on all trees in the search radius sr, except the central tree; SDIci served as a proxy for local density and competition. We used the concept of the stand density index (SDI) (Reineke, 1933). The SDI is a measure of relative density. It provides the stand density in terms of trees per hectare for a stand with an index mean tree diameter of 25 cm. All trees within the circle were used to calculate the local density *n* on circle area *a*. *N* = 10·000/*a* × *n* was the respective tree number upscaled to 1 ha. For the *n* trees, we calculated the quadratic mean stem diameter, dq; based on *N* and dq we then calculated the local density SDI = *N* × (25/dq) around each individual tree. The local SDI was calculated using species-specific allometric exponents derived by Pretzsch and Biber (2005). Note that the exponents *α* were derived on unthinned and A-grade plots of long-term experiments in South Germany that are located in the same area as the age series of this study. On A-grade plots the treatment is restricted to the measurement and removal of dead or dying trees (VDFV, 1902); they serve as reference for the thinned plots. The derived exponents *α* deviated from the species-overarching exponent of −1·605, as proposed by Reineke (1933), are species-specific and representative of South Germany. The resulting competition index SDIci is distant-dependent and easy to interpret. SDIci values were calculated with and without the removed trees for all circles and all surveys; the relationships reported in the Results section were based on the SDI values of the remaining stand at the end of each survey period.

The sampled trees were also used to calculate local mixing proportions. The mixing proportions $m_{1}\ldots m_{n}$ should reflect the area proportions of two or more species in the observed mixed stands (Dirnberger *et al.*, 2017; Pretzsch and del Río, 2020). Tree number, basal area or volume proportions are only appropriate for this purpose if the mixed species have similar growing area requirements (Pretzsch *et al.*, 2017).

To standardize the density and to calculate the unbiased area-related mixing proportions and leaf area indices, we applied the equivalence factors of Pretzsch and Biber (2016). They take into consideration that the analysed species vary *per se* in the growing area requirement and maximum stand density in fully stocked stands. For example, a European beech with a stem diameter of 25 cm may require approximately double the growing space of a Norway spruce of the same diameter; i.e. the density in terms of trees per hectare is only half of that of Norway spruce. The equivalence factors adjust these species-specific differences. Supplementary Data Table S4 gives an overview of the equivalence factors that were applied in this study.

Before calculating the local SDI values and mixing proportions for neighbourhood analysis, we established a toroidal shift of the plot to all eight directions of the plot periphery for edge bias compensation (Radtke and Burkhart, 1998; Pommerening and Stoyan, 2006; Pretzsch, 2009). We use the plot SON 814/2 at the survey in autumn 2011 for visualization of this method in Supplementary Data Fig. S2. Using the toroidal shift, we extended the same mixing patterns and distances between trees in all eight directions and avoided any overestimation of density, which could result from other techniques (Radtke and Burkhart, 1998).

### Structure and growth on the sample circles

In order to compare structure, growth and yield characteristics between mono-specific and mixed parts of our study plots we used the sample circles as described in the section Analysing tree competition and neighbourhood and in Fig. 2B. For all of the ~92 000 sample circles (one circle for each tree and survey) we evaluated the standing volume, mixing proportion, stand density, LAI, above-ground stem mass and mass growth and scaled it up to a hectare [see variables in Table 2, section (ii)]. The SDIc on each circle was calculated analogously to the calculation of SDIci introduced in the section Analysing tree competition and neighbourhood. SDIc was based on all trees on the circle, whereas SDIci was based on all trees on the circle except the central tree. SDIc was calculated for all trees on each circle with and without the removed trees. The LAI for each circle (LAIc) was based on the functions introduced in the section Auxiliary relationships. We further calculated the standing stem volume and above-ground biomass per hectare based on all trees on each circle using the biomass functions in the section Auxiliary relationships. For some rare tree species, we used the generalized functions for conifers and broad-leaved species, respectively. We used the functions by Forrester *et al.*, (2017) for estimating leaf area and biomass; the functions are provided in Supplementary Data Tables S2 and S3. To explore the effects of the codetermining variables on the mixing effects, we introduced dummy variables that indicated the respective species assemblage. Circles with an admixture <10 % of other species based on the SDIc were classified as mono-specific (*m *= 0), and those with an admixture of ≥10 % were classified as mixed (*m *= 1).

This separation was used as it is in line with common definitions (Bravo-Oviedo *et al.*, 2014) of mixed species stands and it split our data in two subgroups with similar sample size. An increase of the threshold from 10 to 15 or 20 % hardly changed the results, however, and increase beyond 20 % obscured the differences between mono- and mixed-species stands. The calculations at the sample circle level resulted in the variables stand density (SDIc), above-ground mass growth (IMc), quadratic mean tree diameter (dqc), diameter growth (id), leaf area index (LAIc) and the categorical variable (*m*). These variables were calculated for both species in each mixture separately but also as sums (e.g. in the case of LAIc) or means (e.g. in the case of dgc) for both species. In order to address the fact that these variables were derived at the sample circle level we attached a ‘c’ to the variable names [see section (2) in Table 2].

### Stand level characteristics

To give an overview of the included age series and their plots, we also evaluated them at the stand level. The stand level characteristics were derived from the successive inventories of the tree diameters and heights, and records of the removal trees. We used standard evaluation methods according to the DESER-norm recommended by the German Association of Forest Research Institutes (in German ‘Deutscher Verband Forstlicher Forschungsanstalten’) (Biber, 2013; Johann, 1993). For estimating the merchantable stem volume in dependence on tree diameter, tree height and form factor, we used the approach by Franz *et al.* (1973) with the stem form equations and coefficients published by Pretzsch (2002, p. 170). The results encompassed the quadratic mean tree diameter, stand volume and volume growth. By applying auxiliary functions (section Auxiliary relationships), we also calculated the LAI and carbon stock for each stand and survey, and the overarching mean, minimum and maximum values for the six species mixtures (see stand variables in Table 2 and Overview of tree and stand characteristics in the Results section).

### Statistical models

To test hypotheses H I to H III, we applied the linear mixed Models 4–10 shown below. The dependent variables were the IMc, LAIc and mean and species-specific id values. The independent variables were individual tree diameter, *d*, quadratic mean diameter within the respective circle (dqc), quadratic mean stem diameter of the stand (dq), the leaf area index LAIc and SDIc on the circle, and the categorical variable *m*, which indicates mono-specific (*m *= 0) and mixed-species conditions (for explanation of variables see Table 2). The diameter *d* indicates the size of the central tree, dqc is the mean tree size in the local environment of the individual tree, dq is the development stage and age of the stand, and the categorical variable *m* indicates whether the subset was mono-specific or mixed.

In all equations, indexes *i* and *k* represent the *k*th observation of the *i*th tree. The fixed effects were covered by parameters *a*_0_ to *a*_n_. With the random effect $b_{i}∼N(0,\;\tau^{2})$, we cover the correlation between the single observations at the tree level. In preliminary model formulations, we also worked with random effects at the plot level; that is, one additional nesting level. As this caused confounding effects with the fixed effects, we constrained ourselves to the simpler random effect structure of eqns (4)–(10). With *ε*_*ik*_, we denoted independently and identically distributed errors.


$$\begin{array}{ll} ln(IMc)= & \;a_{0}+a_{1}\times ln(dqc_{ik})+a_{2}\times m_{i}\\ & +a_{3}\times ln(dqc_{ik})\times m_{i}+b_{i}{+}_{\varepsilon ik} \end{array}$$ (4.1)

$$\begin{array}{l} ln(IMc)=a_{0}+a_{1}\times ln(dqc_{ik})+a_{2}\times m_{i}+b_{i}{+}_{\varepsilon ik} \end{array}$$ (4.2)

$$\begin{array}{l} ln(id)=a_{0}+a_{1}\times ln(dqc_{ik})+a_{2}\times m_{i}+b_{i}{+}_{\varepsilon ik} \end{array}$$ (5)

$$\begin{array}{l} ln(LAIc)=a_{0}+a_{1}\times ln(dqc_{ik})+a_{2}\times ln(dq_{ik})+a_{3}\times m_{i}+b_{i}{+}_{ik} \end{array}$$ (6)

$$\begin{array}{l} ln(IMc)=a_{0}+a_{1}\times ln(LAIc_{ik})+a_{2}\times m_{i}+b_{i}{+}_{\varepsilon ik} \end{array}$$ (7)

$$\begin{array}{ll} ln(id)= & \;a_{0}+a_{1}\times ln(d_{ik})+a_{2}\times ln(dqc_{ik})\\ & +a_{3}\times ln(SDIci_{ik})\times m_{i}+b_{i}{+}_{\varepsilon ik} \end{array}$$ (8)

The de-logarithmic version of the models, for example $y=e^{a_{1}}\times x_{2}^{a_{2}}\times e^{a_{3}\times m}$, shows that the dummy variable m becomes $e^{0}=1$ in the case of monoculture and $e^{a_{3}}$ in the case of mixed stands. This means that $e^{a_{3}}$ directly reveals any multiplicative effects of mixing on the dependent variables. Assuming $a_{3}=0.25$, the mixing effect on the target variable would be $e^{0.25}=1.284$, and the effect would be 28.4 %. This helps to easily interpret the biological meaning of the respective coefficients of *m* in Models 1–8.

To analyse whether the trade-off between stem size growth and stand productivity is modified by tree species mixing, we first fitted the relationships between stem diameter growth and stand density for mono- and mixed-species stands, $id=f_{1}(SDIc,\;m)$, and in the same way the relationships between stand growth and stand density for mono- and mixed-species stands, $IMc=f_{2}(SDIc,\;m)$.


$$\begin{array}{l} ln(id)=a_{0}+a_{1}\times ln(SDIc_{ik})+a_{2}\times m_{i}+b_{i}{+}_{\varepsilon ik} \end{array}$$ (9)

$$\begin{array}{l} ln(IMc)=a_{0}+a_{1}\times ln(SDIc_{ik})+a_{2}\times m_{i}+b_{i}{+}_{\varepsilon ik} \end{array}$$ (10)

Both equations were equalized in terms of ln(SDIc), rearranged, and solved so that IMc was on the left and id on the right side, $IMc=f_{3}(id,\;m)$. By inserting *m *= 0 and *m *= 1, respectively, this resulted in productivity–stem growth relationships for mono-specific and mixed-species stands, respectively. For the step-by-step derivation of eqn (11), see Supplementary Data Derivation A.


$$\begin{array}{l} IMc=e^{b_{1}/b_{2}\times (\ln\left(id-a_{2}\right)+a_{1}-(c_{2}\times b_{1}/b_{2}-c_{1})\times m} \end{array}$$ (11)

**Fig. 3.**
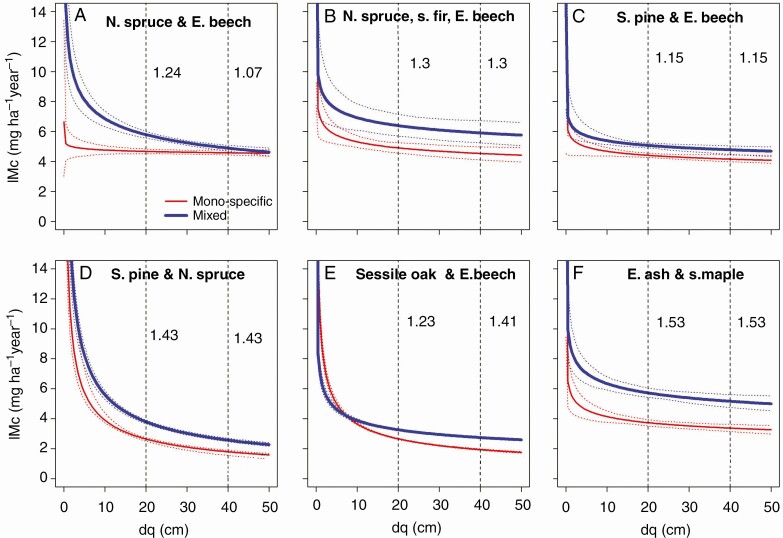
Development of the stand stem mass growth of mixed stands compared with mono-specific stands with increasing quadratic mean stand diameter, dq, as proxy of stand development, shown for (A) Norway spruce and European beech, (B) Norway spruce, silver fir and European beech, (C) Scots pine and European beech, (D) Scots pine and Norway spruce, (E) sessile oak and European beech, and (F) European ash and sycamore maple. The vertical dashed lines and the values (ratios) shown to their right indicate the relationship between mixed and mono-specific stand productivity at a development stage of dq = 20 and 40 cm, respectively. The graphs are based on Model 4 (statistical characteristics are shown in Table 5).

**Fig. 4.**
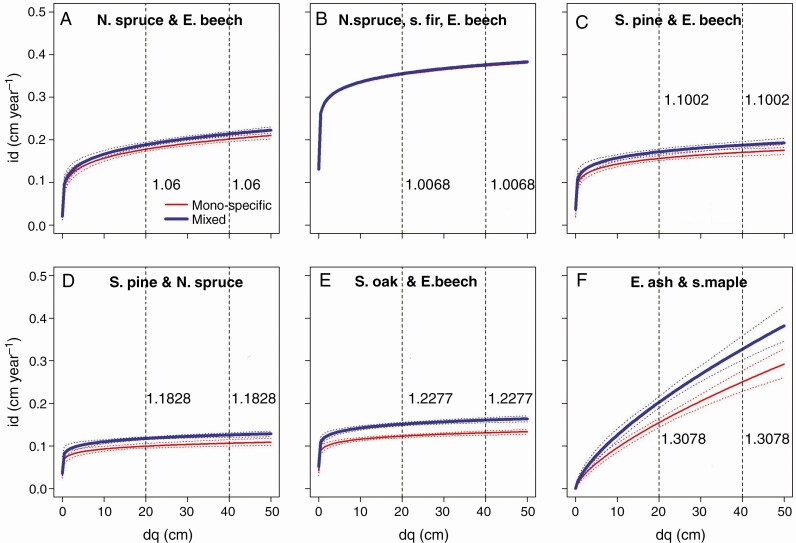
Development of the mean stem diameter increment, id, in mixed compared with mono-specific stands with increasing quadratic mean stand diameter, dq, as proxy of stand development, shown for (A) Norway spruce and European beech, (B) Norway spruce, silver fir, European beech, (C) Scots pine and European beech, (D) Scots pine and Norway spruce, (E) sessile oak and European beech, and (F) European ash and sycamore maple. The id value reflects the mean of both tree species. The vertical dashed lines and the values (ratios) shown to their right indicate the relationship between mixed and mono-specific stand diameter increment at a development state of dq = 20 and 40 cm, respectively. The graphs are based on Model 5 (statistical characteristics are shown in Table 5).

**Fig. 5.**
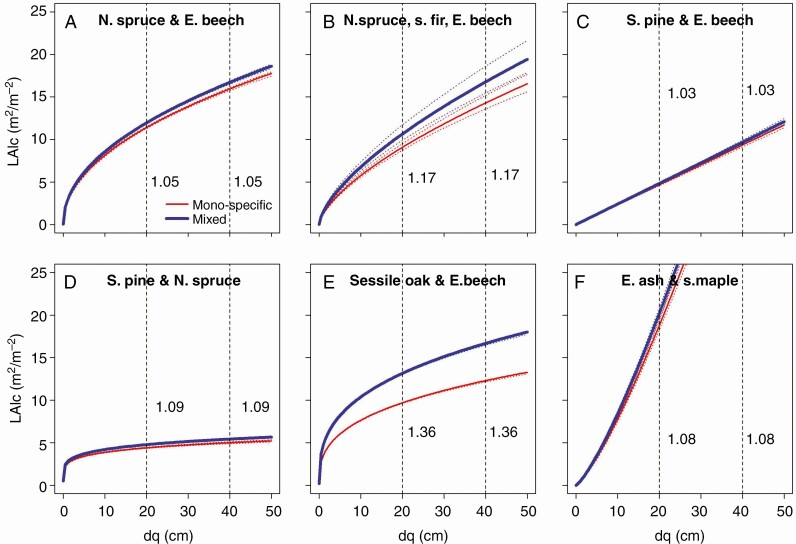
Development of the local leaf area index, LAIc, with increasing quadratic mean stand diameter, dq, as proxy of stand development phase shown for mixed (blue line) compared with mono-specific stands (red line), shown for (A) Norway spruce and European beech, (B) Norway spruce, silver fir, European beech, (C) Scots pine and European beech, (D) Scots pine and Norway spruce, (E) sessile oak and European beech, and (F) European ash and sycamore maple. The vertical dashed lines and the values (ratios) shown to their right indicate the relationship between mixed and mono-specific stand productivity at a development state of dq = 20 and 40 cm, respectively. The graphs are based on Model 6 (statistical characteristics are shown in Table 5).

**Fig. 6.**
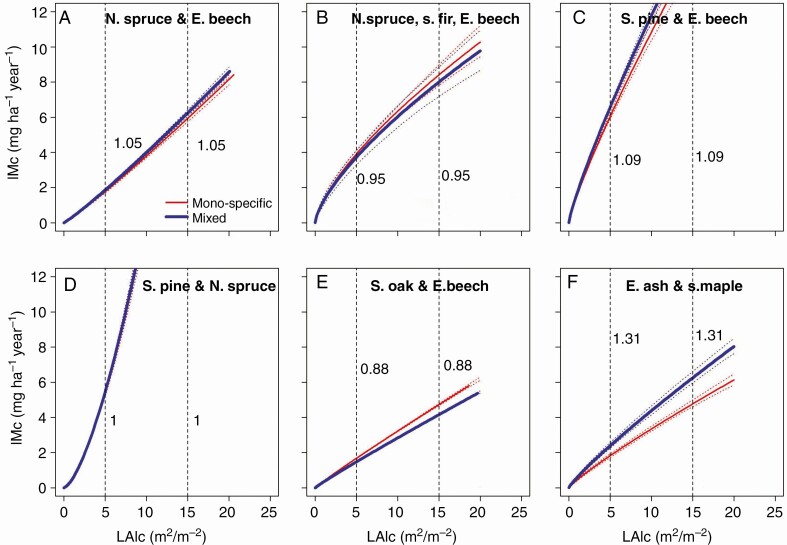
Development of the stand stem mass growth of mixed compared with mono-specific stands depending on stand density in terms of stand leaf area, LA, shown for (A) Norway spruce and European beech, (B) Norway spruce, silver fir, European beech, (C) Scots pine and European beech, (D) Scots pine and Norway spruce, (E) sessile oak and European beech, and (F) European ash and sycamore maple. The vertical dashed lines and the values (ratios) shown to their right indicate the relationship between mixed and mono-specific stand productivity LAI = 5 and 15 m^2^ m^2^, respectively. The graphs are based on Model 7 (statistical characteristics are shown in Table 5).

**Fig. 7.**
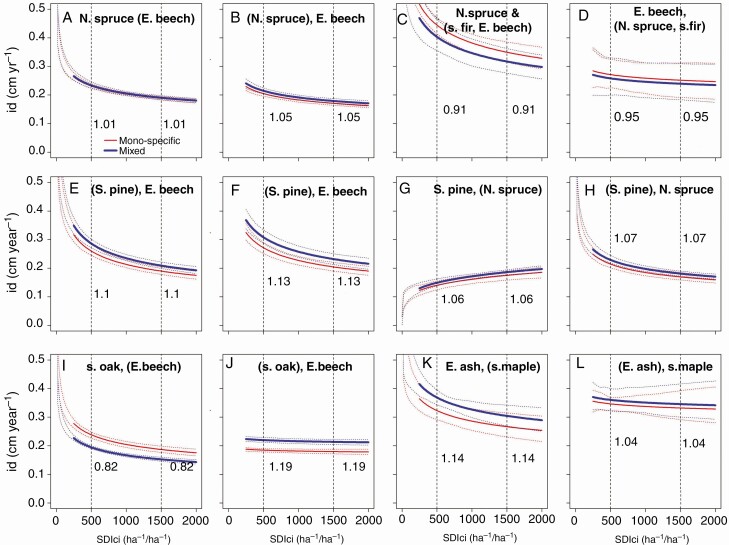
Species-specific dependency of stem diameter increment on stand density in mixed compared with mono-specific stands, shown for (A, B) Norway spruce and European beech, (C, D) Norway spruce, silver fir and European beech, (E, F) Scots pine and European beech, (G, H) Scots pine and Norway spruce, (I, J) sessile oak and European beech, and (K, L) European ash and sycamore maple. The vertical dashed lines and the values (ratios) shown to their right indicate the relationship between stem increment in mixed to mono-specific stands for local SDI values of 500 and 1500 trees per hectare. The visualization was based on Model 8, and the mean diameters were set to 25 cm. N. spruce (E. beech) addresses the behaviour of Norway spruce in mixture with European beech. (N. spruce), E. beech addresses the growth of European beech in mixture with Norway spruce. This nomenclature was used for the other mixtures analogously.

The 95 % confidence intervals displayed with the model predictions in the Results section were derived from bootstrapped model predictions for each combination of input variable values used in the diagrams. For the bootstrapping procedure, we used the function bootMer from the R-Package lme4 (Bates *et al.*, 2015). Even though the confidence bands in Figs 3–7 partly overlapped, the visualized differences between curves for mixed and mono-specific stands resulted from effects that were clearly identified as significant in the mixed model regression. The confidence bands for mixed and mono-specific stands in the same diagram must not be seen independently; that is, an upward deviation relative to the mono-specific curve would mean an upward deviation relative to the mixed stand curve. In other words, one such diagram does not show two independently fitted regression models, but a differentiated view of the same model. For all calculations, we used the statistical software R 3.6.3, and we used the libraries nlme (Pinheiro *et al.*, 2021) and lme4 (Bates *et al.*, 2015).

**Table 5.** Statistical characteristics of the main models used in this study. The equation numbers refer to the models introduced in the Statistical models section. For limitations of space, the table reports only the fixed-effect variables of the respective models. In column “Variables IMc~ dq, m addresses the relationship between IMc and and m. This nomenclature applies for the other relationship analogously. For explanation of variables see footnote of Table 1

Equation	Group	Variables	*n*	*a*_0_	s.d. (*a*_0_)	*P*-value	*a*_1_	s.d. (*a*_1_)	*P*-value	*a*_2_	s.d. (*a*_2_)	*P*-value	*a*_3_	s.d. (*a*_3_)	*P*-value
4.1	N. sp., E. be.	IMc~ dq, *m*	20755	1·6273	0·1079	<0·001	−0·0290	0·0329	<0·3781	0·8599	0·1449	<0·001	−0·2149	0·0439	<0·001
4.2	N. sp., s. fir, E. be.	IMc~ dq, *m*	1521	1·9347	0·1166	<0·001	−0·1148	0·0420	<0·001	0·2645	0·0683	<0·001			
4.2	N. sp., S. pi.	IMc~ dq, *m*	12256	2·6578	0·0905	<0·001	−0·5624	0·0296	<0·001	0·3609	0·0230	<0·001			
4.2	S. pi., E. be.	IMc~ dq, *m*	11517	1·7428	0·0778	<0·001	−0·0865	0·0255	<0·001	0·1402	0·0196	<0·001			
4.1	S. oak, E. be.	IMc~ dq, *m*	24824	2·3473	0·0349	<0·001	−0·4570	0·0117	<0·001	−0·4104	0·0602	<0·001	0·2054	0·0197	<0·001
4.2	E. ash, syc. map.	IMc~ dq, *m*	3279	1·7640	0·1522	<0·001	−0·1489	0·0491	<0·001	0·4270	0·0420	<0·001			
5	N. sp., E. be.	id~ dqc, dq, *m*	16611	−2·2701	0·0891	<0·001	0·1813	0·0266	<0·001	0·05827	0·0156	<0·001			
5	N. sp., s. fir, E. be.	id~dqc, dq, *m*	1420	−1·2868	0·1306	<0·001	0·0819	0·0467	<0·001	0·0068	0·0751	<0·001			
5	N. sp., S. pi.	id~dqc, dq, *m*	9398	−2·5987	0·0928	<0·001	0·0968	0·0296	<0·001	0·1679	0·0244	<0·001			
5	S. pi., E. be.	id~dqc, dq, *m*	9617	−2·2357	0·0913	<0·001	0·1263	0·0296	<0·001	0·0955	0·0214	<0·001			
5	S. oak, E. be	id~dqc, dq, *m*	20205	−2·3588	0·0442	<0·001	0·0880	0·0145	<0·001	0·2052	0·0140	<0·001			
5	E. ash, syc. map.	id~dqc, dq, *m*	2731	−3·9440	0·1718	<0·001	0·6937	0·0543	<0·001	0·2684	0·0442	<0·001			
6	N. sp., E. be.	LAIc~dqc, dq, *m*	20755	0·9845	0·0294	<0·001	1·1332	0·0180	<0·001	−0·6494	0·0200	<0·001	0·0471	0·0052	<0·001
6	N. sp., s. fir, E. be.	LAIc~dqc, dq, *m*	1521	0·2393	0·0642	<0·001	0·6562	0·0227	<0·001	0·1599	0·0352	<0·001			
6	N. sp., S. pi.	LAIc~dqc, dq, *m*	12256	0·9290	0·0272	<0·001	1·5342	0·0168	<0·001	−1·3499	0·0176	<0·001	0·0820	0·0072	<0·001
6	S. pi., E. be.	LAIc~dqc, dq, *m*	11517	−1·4635	0·0311	<0·001	1·5742	0·0191	<0·001	−0·5711	0·0196	<0·001	0·0329	0·0065	<0·001
6	S. oak, E. be	LAIc~dqc, dq, *m*	24824	1·2427	0·0160	<0·001	1·2016	0·0105	<0·001	−0·8581	0·0102	<0·001	0·3050	0·0048	<0·001
6	E. ash, syc. map.	LAIc~dqc, dq, *m*	3279	−0·8704	0·0627	<0·001	2·0059	0·0364	<0·001	−0·7368	0·0338	<0·001	0·0729	0·0167	<0·001
7	N. sp., E. be.	IMc~LAIc, *m*	20755	−1·2060	0·0470	<0·001	1·1026	0·0182	<0·001	0·0497	0·0155	<0·001			
7	N. sp., s. fir, E. be.	IMc~LAIc, *m*	1521	0·2407	0·0739	<0·001	0·6973	0·0352	<0·001	−0·0497	0·0565	<0·379			
7	N. sp., S. pi.	IMc~LAIc, *m*	12256	0·7381	0·0280	<0·001	1·5081	0·0189	<0·001	0·0025	0·0196	<0·001			
7	S. pi., E. be.	IMc~LAIc, *m*	11517	0·4374	0·0249	<0·001	0·8446	0·0170	<0·001	0·0834	0·0171	<0·001			
7	S. oak, E. be	IMc~LAIc, *m*	24824	−1·0045	0·0166	<0·001	0·9444	0·0073	<0·001	−0·1282	0·0072	<0·001			
7	E. ash, syc. map.	IMc~LAIc, *m*	3279	−0·8101	0·0714	<0·001	0·8759	0·0280	<0·001	0·2702	0·0356	<0·001			
8	N. sp., (E. be.)	id~*d*, SDIci, *m*	9936	−2·7460	0·1686	<0·001	0·7589	0·0254	<0·001	−0·1869	0·0213	<0·001	0·0114	0·0195	<0·560
8	(N. sp.), E. be.	id~*d*, SDIci, *m*	6206	−3·7709	0·1780	<0·001	0·9980	0·0296	<0·001	−0·1652	0·0217	<0·001	0·0464	0·0242	<0·050
8	N. sp., (s. fir, E. be.)	id~*d*, SDIci, *m*	1123	−0·8046	0·2092	<0·001	0·4199	0·0422	<0·001	−0·2185	0·0298	<0·001	−0·0975	0·0884	<0·270
8	N. sp., s. fir, E. be.	id~*d*, SDIci, *m*	206	−2·5526	0·7979	<0·001	0·5208	0·1487	<0·001	−0·0689	0·0843	<0·415	−0·0500	0·1575	<0·751
8	N. sp., S. pi.	id~*d*, SDIci, *m*	3576	−4·8825	0·3307	<0·001	0·5192	0·0397	<0·001	0·2007	0·0418	<0·001	0·0611	0·0537	<0·226
8	N. sp., S. pi.	id~*d*, SDIci, *m*	5788	−4·5425	0·1751	<0·001	1·3464	0·0290	<0·001	−0·2142	0·0231	<0·001	0·0658	0·0296	<0·027
8	S. pi., E. be.	id~*d*, SDIci, *m*	3762	−1·2316	0·2196	<0·001	0·5162	0·0373	<0·001	−0·2860	0·0297	<0·001	0·0966	0·0337	<0·004
8	S. pi., E. be.	id~*d*, SDIci, *m*	3554	−2·9990	0·2011	<0·001	1·0253	0·0367	<0·001	−0·2582	0·0269	<0·001	0·1260	0·0332	<0·001
8	S. oak, E. be	id~*d*, SDIci, *m*	8173	−1·9319	0·1628	<0·001	0·5833	0·0178	<0·001	−0·2219	0·0214	<·001	−0·2037	0·0235	<0·001
8	s. oak, E. be	id~*d*, SDIci, *m*	11098	−5·0467	0·0907	<0·001	1·0927	0·0144	<0·001	−0·0258	0·0125	<0·038	0·1740	0·0157	<0·001
8	E. ash, syc. map.	id~*d*, SDIci, *m*	709	−3·6485	0·3790	<0·001	1·1182	0·0564	<0·001	−0·1739	0·0572	<0·002	0·1322	0·0594	<0·027
8	E. ash, syc. map.	id~*d*, SDIci, *m*	1290	−5·7095	0·4062	<0·001	1·5194	0·0483	<0·001	−0·0388	0·0576	<0·500	0·0398	0·0582	<0·495
9	N. sp., E. be.	id~SDIc, *m*	16611	−0·5153	0·1078	<0·001	−0·1690	0·0156	<0·001	0·0920	0·0158	<0·001			
9	N. sp., s. fir, E. be.	id~SDIc, *m*	1420	−0·0578	0·1772	<0·001	−0·1663	0·0287	<0·001	0·1294	0·0722	<0·071			
9	N. sp., S. pi.	id~SDIc, *m*	9398	−1·2340	0·1392	<0·001	−0·1618	0·0202	<0·001	0·2209	0·0254	<0·001			
9	S. pi., E. be.	id~SDIc, *m*	9617	−0·2529	0·1213	<0·037	−0·2440	0·0182	<0·001	0·1463	0·0215	<0·001			
9	S. oak, E. be.	id~SDIc, *m*	20205	−0·9649	0·0744	<0·001	−0·1832	0·0117	<0·001	0·2492	0·0138	<0·001			
9	E. ash, syc. map.	id~SDIc, *m*	2731	−0·8018	0·2556	<0·002	−0·1615	0·0411	<0·001	0·2705	0·0460	<0·001			
10	N. sp., E. be.	IM~SDIc, *m*	20755	−3·1854	0·1057	<0·001	0·6883	0·0153	<0·001	0·0563	0·0160	<0·001			
10	N. sp., s. fir, E. be.	IM~SDIc, *m*	1521	−1·0519	0·1391	<0·001	0·4409	0·0225	<0·001	0·0329	0·0565	<0·561			
10	N. sp., S. pi.	IM~SDIc, *m*	12256	−5·0963	0·1268	<0·001	0·9000	0·0187	<0·001	0·0788	0·0227	<0·001			
10	S. pi., E. be.	IM~SDIc, *m*	11517	−2·2157	0·1041	<0·001	0·5567	0·0156	<0·001	0·0703	0·0182	<0·001			
10	S. oak, E. be.	IM~SDIc, *m*	24824	−3·6260	0·0353	<0·001	0·7481	0·0056	<0·001	0·0251	0·0069	<0·001			
10	E. ash, syc. map.	IM~SDIc, *m*	3279	−4·2795	0·2086	<0·001	0·8999	0·0334	<0·001	0·3350	0·0369	<0·001			

## RESULTS

### Overview of tree and stand characteristics

Table 4 shows the mean stand characteristics over all surveys and substantiates that all mixtures are represented by young to old stands. For further evaluation, we used the mean tree diameter as a substitute for stand age, as in practice it is easier for access. The stand volume was the highest (1774 m^3^ ha^−1^) in the mature stands of Norway spruce and European beech. The LAI was higher in mixtures with shade-tolerant species than in those with light-demanding species. The carbon storage in the above-ground stem mass was the highest (388 mg ha^−1^) in old Norway spruce/European beech stands. Annual stem volume growth was the highest in Scots pine/European beech, Norway spruce/European beech and Scots pine/Norway spruce stands.

**Table 4.** Overview of some characteristics of the study stands shown separately for the six tree species mixtures. The table shows the mean, standard deviation, minimum and maximum of the stand age, quadratic mean stem diameter (dq), stand stem volume (*V*), LAI, above-ground carbon content (*C*) and mean annual stem volume increment (IV) of the stands of all surveys

Variable	Unit	Mean	s.d.	Minimum	Maximum
Norway spruce and European beech					
Stand age	years	79·9	28·3	36·0	138·0
dq	cm	32·1	10·4	14·3	51·5
*V*	m^3^ ha^−1^	684·5	268·5	172·0	1774·0
LAI	m m^−1^	10·2	2·6	5·6	24·2
*C*	mg ha^−1^	160·9	48·0	63·5	388·2
IV	m^3^ ha^−1^ yr^−1^	17·7	4·7	7·3	27·7
Sessile oak and European beech					
Stand age	years	81·2	42·3	17·0	238
dq	cm	23·2	9·2	7·8	47·6
*V*	m^3^ ha^−1^	443·4	177·7	72·0	774·0
LAI	m m^−1^	10·1	2·0	6·2	13·7
*C*	mg ha^−1^	163·8	63·2	46·6	295·5
IV	m^3^ ha^−1^ yr^−1^	13·6	2·6	7·9	18·8
Scots pine and European beech					
Stand age	years	77·7	51·5	15·0	214·0
dq	cm	22·2	7·4	10·2	36·7
*V*	m^3^ ha^−1^	490·4	255·6	120·0	1063·0
LAI	m m^−1^	6·5	2·4	2·6	12·8
*C*	mg ha^−1^	115·8	43·6	43·7	209·3
IV	m^3^ ha^−1^ yr^−1^	18·4	6·1	10·0	36·2
Norway spruce, silver fir and European beech					
Stand age	years	147·3	64·9	43	218
dq	cm	26·3	6·5	15·2	37·2
V	m^3^ ha^−1^	457·3	129·3	279·0	672·0
LAI	m m^−1^	8·5	2·5	5·4	12·6
C	mg ha^−1^	117·5	27·9	75·4	167·2
IV	m^3^ ha^−1^ yr^-1^	11·9	1·0	11·2	13·1
European maple and European ash					
Stand age	years	56·44	21·9	20	116
dq	cm	20·5	7·2	8·1	34·2
*V*	m^3^ ha^−1^	356·0	153·6	76·0	656·0
LAI	m m^−1^	5·7	1·4	3·3	9·0
*C*	mg ha^-1^	104·1	33·9	39·7	188·5
IV	m^3^ ha^−1^ yr^−1^	15·9	2·1	10·4	18·6
Scots pine and Norway spruce					
Stand age	years	74·7	32·1	22·0	118·0
dq	cm	23·3	8·1	11·4	36·7
*V*	m^3^ ha^−1^	625·7	205·4	256·0	918·0
LAI	m m^−1^	7·2	1·1	4·9	8·6
*C*	mg ha^−1^	128·7	29·5	79·2	171·3
IV	m^3^ ha^−1^ yr^−1^	19·8	3·9	14·4	25·8

### Stand and tree growth in mixed versus mono-specific stands throughout the rotation (H I)

For all mixtures, we found a decrease in stand stem mass growth, IMc, with increasing quadratic mean tree diameter, which we used as a proxy for stand age. Regarding H I, we found that, in all cases, mixed stands were more productive in terms of stand stem mass growth than mono-specific stands throughout the entire rotation. In the stand development phase of dq = 20 cm, we found an overyielding in terms of stand stem mass of 15–53 %, and at the stand phase of dq = 40 cm an overyielding of 7–53 % (Fig. 3). In only one case, the mixture Norway spruce/European beech, overyielding decreased with age (Fig. 3A), and in all other cases we found a constancy or even an increase in overyielding (Fig. 3B–F). The superiority continued until the advanced stand development phases. Interestingly, we also found high overyielding for species combinations with rather similar physiological traits, for example in stands with shade-tolerant mixtures (Norway spruce, silver fir, European beech; 30 %) or light-demanding mixtures (European ash, sycamore maple, 53 %).

Except for the mixture of Norway spruce, silver fir and European beech, the mean stem diameter growth of both species was always higher in mixed stands than in mono-specific stands (Fig. 4) throughout the whole rotation. We found no significant change in this effect with progressing stand development, i.e. there were no significant interactions between factors *m* and dq in Model 5 (Table 5). Referring to the index diameters of dq = 20 and dq = 40 cm (vertical dashed lines in Fig. 4), the superiority remained the same. The superiority ranged from 6 % in Norway spruce and European beech to 31 % in European ash and sycamore maple. Note that this analysis only distinguished between mono-specific (mixing portion < 10%) and mixed-species conditions (mixing portion ≥10 %); future studies may analyse the impact of different mixing proportions.

### Modified stand density and growth efficiency of mixed versus mono-specific stands throughout the rotation (H II)

H II refers to two different potential causes of overyielding, i.e. an increased stand density that may increase productivity by higher packing density of trees and leaf area, and an increased growth efficiency of a given density or leaf area. Figure 5 shows, for all six considered species combinations, a higher stand leaf area in mixed compared with the mean of the mono-specific stands. The visualization of the results is based on Model 6 and the respective coefficients are shown in Table 5. In order to demonstrate the magnitude of overdensity and any changes with progressing stand development, the figures indicate the ratios $LAIc_{mix}/LAIc_{mono}$ for stands with dq = 20 and 40 cm (dashed vertical lines with added ratios). In all mixtures the overdensity of mixed stands is maintained throughout the whole rotation ($LAIc_{mix}/LAIc_{mono}>1\cdot 0$). The overdensity ranged between 3 % in case of Scots pine/European beech and 36% in mixed stands of sessile oak and European beech. We found no decrease or increase of the overdensity with progressing stand development.

Regarding the growth efficiency per given stand density and leaf area (Fig. 6), we found a less general effect of mixing. In half of the considered mixtures, leaf area efficiency hardly increased (Scots pine and Norway spruce) or even reduced (sessile oak and European beech). In the other cases, the growth efficiency of the leaf area increased by 5–31 %. In summary, we found that the increased productivity was mainly a density effect in the case of sessile oak and European beech, mainly an efficiency effect in the case of European ash and sycamore maple, and a combination of both in the other species assemblages.

### Reduction of competition and attenuation of the relationship between stand density and stem growth (H III)

Under the same competition index, SDIci tree diameter increment was higher in mixed stands than in mono-specific stands in 9 out of 12 cases (Fig. 7A–L). Figure 7 visualizes for each of the six analysed species combinations and for both species of the respective mixtures how they grow in mixed (blue) compared with mono-specific (red) neighbourhood. For example, Fig. 7A shows that in the combination Norway spruce/European beech, the growth of Norway spruce in mixture (+ 1%) hardly differs from its growth in mono-specific stands. The superiority of the growth in mixture ranged from 1 to 19 %. N. spruce (E. beech) addresses the behaviour of Norway spruce in mixture with European beech. (N. spruce), E. beech addresses the growth of European beech in mixture with Norway spruce. This nomenclature was used for the other mixtures analogously. Norway spruce and European beech in a mixture of Norway spruce, silver fir and European beech (Fig. 7C, D) and sessile oak in mixture with European beech (Fig. 7I) grew less under similar competition. With the exception of Scots pine when mixed with Norway spruce (Fig. 7G), the stem diameter growth, id, always decreased with increasing stand density.

In the majority of cases, the growth efficiency was increased by tree species mixing. This again indicates that there were no overall species-specific patterns and that both density and efficiency effects needed to be considered for understanding, modelling or silvicultural steering.

**Fig. 8.**
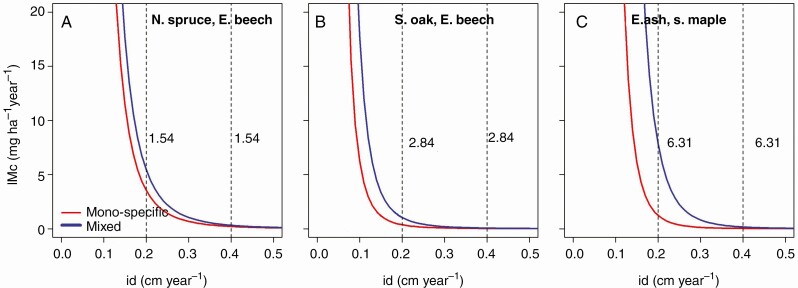
Trade-off between stand mass growth and stem diameter increment in mixed compared with mono-specific stands, shown by example for the mixture of (A) Norway spruce and European beech, (B) sessile oak and European beech, and (C) European ash and sycamore maple. Analogous relationships for the other considered species mixture are shown in Supplementary Data Fig. S3.

Figure 8 shows the trade-off between stand productivity and stem growth in mono- and mixed-species stands. For this purpose, we equalized in terms of ln(SDIc) the relationships between mean stem diameter increment and SDIc (Table 5, Model 9) and the relationship between stand mass growth and SDIc (Table 5, Model 10) [eqn (11) and Supplementary Data Derivation A].

Figure 8 shows how much of stand productivity IMc is required to increase the mean tree diameter by stand density reduction in mono-specific stands (red lines), and how the relationship changes in mixed stands (blue lines). As we found similar patterns in all six species assemblages we restricted the visualization of the results to three mixtures (see Supplementary Data Fig. S3 for the other three mixtures). It is known from other studies of mono-specific stands that stem diameter increment decreases with increasing stand density. The red hyperbolas in Fig. 8 reflect this well-known trade-off. Interestingly, tree species mixing attenuates this relationship; similar tree diameter growth can be achieved under higher density and higher stand productivity. This means that a higher density level comes along with higher size growth and taller trees. The ratios shown in Fig. 8 indicate that a stem diameter increment of 2 mm, for example, can be achieved with much higher stand density in mixed compared with mono-specific stands, with the ratios $IMc_{mix}/IMc_{mono}\;$ranging between 1·5 and 6·31 (Fig. 8 and Supplementary Data Fig. S3).

## DISCUSSION

### Increased stand productivity and stem diameter growth throughout the whole rotation (H I)

As our study was based on 11 age series and 63 plots in stands with mixed and mono-specific parts with an age range of 22–238 years, we arrived at substantial information about the continuation of overyielding and diameter growth increase with progressing stand development. Whereas most studies so far have dealt with young- or medium-aged stands, our results provide evidence that overyielding in terms of stem mass growth of 7–53 % can continue over the whole rotation (Fig. 3). Overyielding slightly abated when plotted over quadratic mean tree diameter (species combination of Norway spruce and European beech; Fig. 3A), staying constant (in most cases; Fig. 3–D, F) or slightly increasing (sessile oak/European beech; Fig. 3E). A mean overyielding of 1–2 mg ha^−1^ year^−1^ throughout the whole rotation accumulates to a plus of 100–200 mg ha^−1^, which is a plus of ~200–400 m^3^ ha^−1^ in the case of a rotation time of 100 years. Longer rotation times, as usual for sessile oak/European beech or Scots pine/European beech, result in even higher accumulated amounts of overyielding.

We found overyielding similar to the magnitude reported by Pretzsch *et al*. (2010, 2015, 2020), Thurm and Pretzsch (2016), Jactel *et al.* (2018), Steckel *et al.* (2019) and Ruiz-Peinado *et al.* (2021). Similar to Kelty (1992) and Jactel *et al.* (2018), we found that mixtures within conifers and broad-leaved species and between these two groups achieved significant overyielding. The superiority of stem diameter growth (Fig. 4) ranged from 6 % in Norway spruce and European beech to 31 % in European ash and sycamore maple, and continued throughout the rotation.

We based the analyses on stem mass growth as the biomass yield is most informative for production ecology (Landsberg, 1986; Kelty, 1992) and in order to eliminate any overestimation of overyielding due to differences in the species’ wood densities (Knigge and Schulz, 1966; Zeller *et al.*, 2017). Especially in mixtures with conifers of relatively low wood densities (e.g. Norway spruce, Scots pine; 0.3–0.4 g cm^−3^), the overyielding in terms of volume growth can be 1–5 % higher when mixed with species with higher wood density (e.g. European beech, European beech; 0.4–0.6 g cm^−3^).

When comparing the stand and tree traits between mixed and mono-specific stands, we used the quadratic mean tree diameter of the stand as a proxy for the stand development phase. The results were analogous when stand age (Table 4) was used as a predictor. However, stem diameter is more feasible for silvicultural guidelines and forest management, as they commonly use the mean stem dimensions (e.g. stem diameter, height or volume) as measures and criteria for scheduling silvicultural interventions (Schober, 1987; Abetz, 1974, 1988; Newton, 1997; Bégin *et al.*, 2001).

### Increased stand density and growth efficiency determine mixing effects (H II)

In this study, we did not analyse the mechanisms of species interactions; however, the reaction pattern in terms of stand density and the increase in growth efficiency as a result of mixing hypotheses about the underlying causes. The strong increase in stand density that resulted in significant overyielding of all mixtures was probably promoted by species complementarity in space occupation.

Species complementarity in structure and function has been repeatedly discussed as relevant for competition reduction, density increase and overyielding (Barbeito *et al.*, 2017; Juchheim *et al.*, 2017; Zeller *et al.*, 2021). Other studies have found that stand density was higher in terms of basal area and SDI (Williams *et al.*, 2017; Thurm and Pretzsch, 2021), crown projection area (Pretzsch, 2014) or LAI (Peng *et al.*, 2017) in mixed compared with mono-specific stands. The different species-specific crown allometry may enable a higher packing density of crowns (Jucker *et al.*, 2015). In addition, mixing may modify crown shyness and mechanical abrasion (Fish *et al.*, 2006; Hajek *et al.*, 2015). Meng *et al.* (2006) showed that the prevention of crown collisions by fixing trees with ropes can increase the crown cover and leaf area of mature stands. Crown shyness may be reduced and leaf area increased if the mechanical abrasion is reduced as species occupy different layers and have their maximal lateral extension (Barbeito *et al.*, 2017). Differences in light ecology may enable a richer vertical layering and structuring; for example, European beech may easily survive under Scots pine because of its higher shade tolerance and lower light compensation point (Ellenberg and Leuschner, 2010). All this may contribute to overyielding via increased packing density in the inter-specific neighbourhood.

We used leaf area equations (la = f(*d*) relationships) that were mainly based on mono-specific stands (Forrester *et al.*, 2017). As crowns of a given stem diameter can be larger and more plastic in mixed compared with mono-specific stands (Barbeito *et al.*, 2017; Pretzsch, 2019) and can have higher crown projection and leaf area (Thurm and Pretzsch, 2016), the reported relative superiority may even be underestimated. As we always chose tree positions as the midpoint of the constructed sampling circles (Fig. 2), the absolute leaf area estimation may have a positive bias. This may be caused by the fact that the centre of the plot is certainly always covered by the central tree, so canopy coverage and leaf area are over-represented. By a selection of random positions as circle midpoints, also locations without trees and shading leaf area would have been included. This would probably have resulted in lower values of the stand characteristics. However, we applied the same sampling procedure (concentric circles around the tree positions) in the mixed and mono-specific parts of the plots. This means that the absolute values might be overestimated; however, the comparison between mixed and mono-specific sample circles, both equally biased, should result in reliable relationships between the two groups. Potential sources of errors by using auxiliary functions were kept as small as possible. For this purpose, we only applied functions for height and leaf area estimation and allometric exponents and equivalence factors that were derived for the studied tree species, the included stands or the study region.

We used generalized equivalence factors for calculating appropriate area-related mixing proportions for the species with different growing space requirements. Alternative approaches would have been the use of yield tables (Kramer und Akça, 1987, p. 187), wood dry mass relationships between species (Assmann, 1970, pp. 360–361) or leaf area measurements on the plots (Dirnberger *et al.*, 2017). However, the common yield tables seemed to be too outdated for this purpose, the use of wood dry mass is questionable due to dependency of the specific gravity on the respective stand density management, and measurements of the leaf area in the stands were not available. Therefore, we applied the density equivalence factors that were derived for this purpose from stands in the actual study region (Pretzsch and Biber, 2016) and that have already been successfully applied in previous studies (del Río et al., 2016; Pretzsch and del Río, 2020). For analysing the effect of mixing on the stand density and growth efficiency (Figs 4 and 5) we used the LAI as the density measure in order to keep the link to forest ecology, tree eco-physiology and remote sensing. Stand basal area, standing stem volume and SDI are proven dendrometric measures for stand density but hardly known beyond forest growth and yield science. However, the SDI as density measure was available for all trees and sample circles and yielded similar effects of tree species mixing on stand density and growth. The increase in growth efficiency suggests a facilitation effect in inter-specific neighbourhoods as repeatedly reported, especially on harsh sites and in dry years (Callaway, 1998; Pretzsch *et al.*, 2013; del Río *et al.*, 2014). Trees with the same diameter were more efficient in mixed than in mono-specific stands despite the higher stand density, which suggests an efficiency increase of the leaf area and resources use. An increase in efficiency means that at lower densities an inter-specific neighbour may increase the efficiency of the crown, similar to a fertilization effect (Khanna, 1997). In this case, benefits may emerge independently of stand density, but also under wide spacing and strong thinning. The facilitation effects on our plots may be caused by hydraulic lift (Zapater *et al.*, 2011; Steckel *et al.*, 2019), improvement of nutrients (Rothe and Binkley, 2001; Augusto *et al.*, 2002; Jonard *et al.*, 2008) and light supply (Forrester *et al*., 2018, 2019).

### Reduction of competition and attenuation of stand density–stem growth trade-off (H III)

The acceleration of stem diameter growth to achieve taller trees in a shorter time or to reduce rotation requires stand density reduction by thinning (Zeide, 2001; Pretzsch, 2020). Density reductions stronger than moderate thinning cost stand growth (Assmann, 1970). These relationships are well known from numerous thinning trials in mono-specific stands (O’Hara, 1988; Juodvalkis *et al.*, 2005), well integrated into growth models (Courbaud *et al.*, 2001; Franklin *et al.*, 2009) and reflected by silvicultural guidelines (Pelletier and Pitt, 2008). This study showed that mixture can significantly attenuate the trade-off between individual stem diameter growth and stand growth (Fig. 8). Similar growth rates may be achieved under higher density and higher stand productivity; *ceteris paribus*, they require lower losses of stand productivity. On the other hand, a higher density level resulted in higher size growth and taller trees. These findings indicate that the mitigation potential of forests by production of highly dimensioned and long-lived forest products becomes possible with lower expense on stand stock and mass productivity at the stand level. In this way, mixed stands may increase both the adaptation to climate change risks and the mitigation effect of higher carbon storage. To implement such findings, they should be integrated into silvicultural guidelines for mixed species stands, as claimed by Coll *et al.* (2018) and proposed by Pretzsch and Zenner (2017) and Mason *et al.* (2018).

### Consequences for measurement and modelling mixed-species stands

This study showed that species-specific structure and composition strongly co-determine the density and productivity of the stands and the growth of individual trees. In the past, the structural properties of the stands have been measured mainly manually (e.g. using measuring tape, crown mirror, theodolite), and the structural information of mixed stand analyses can be currently obtained more efficiently by terrestrial LiDAR (T-LiDAR) (Olivier *et al.*, 2016; Juchheim *et al.*, 2017). T-LiDAR, especially, can provide key information such as tree size and mass (Puletti *et al.*, 2020), crown characteristics (Barbeito *et al.*, 2017; Jacobs *et al.*, 2020), spatial stand structure (Bayer *et al.*, 2013, Bayer and Pretzsch, 2017; Beyer *et al.*, 2017, del Río *et al.*, 2017), species identity (Terryn *et al.*, 2020) and leaf area (Soma *et al.*, 2020) and crown transparency (Jacobs *et al.*, 2021) more easily than in the past. Methods for measuring leaf area, stand structure and species identification are occasionally used in forest and ecological science (Dassot *et al.*, 2011) are in preparation for standard application and will get much easier access to exactly those tree and stand characteristics that make the difference between mixed and mono-specific stand dynamics and performance (Lintunen *et al.*, 2011).

The finding that the species combination within a tree neighbourhood strongly determines tree and stand growth underlines the need for spatially dependent model approaches and species-specific parameterization. Most of these models are based on a potential growth rate that is continuously reduced with increasing competition (potential modifier approach; Pretzsch *et al*., 2002, 2015). The finding that mixing can increase the stand density, reduce the competition and significantly raise the growth beyond the growth level in mono-specific stands suggests that the commonly used inverse J-shaped potential modifier function should be reconsidered. Maybe it should be replaced by a unimodal-shaped function with a maximum growth rate not under solitary and mono-specific conditions but under low density and in an inter-specific neighbourhood. We suggest that the level and shape of the potential modifier functions in individual tree models need to be adapted in order to take into consideration the beneficial effects of competition reduction and facilitation. In addition, competition and mortality models need to be adapted to consider the mixing effects on competition and density. Tree-level-based models have the advantage that they can deduce the reaction patterns for the whole continuum of mono-specific and mixed species stands mechanistically from tree–tree interactions (Cole and Lorimer, 1994; Maguire *et al.*, 1998; Pretzsch *et al.*, 2002) and can consider both density and efficiency effects (Thorpe *et al.*, 2010; Bravo *et al.*, 2019).

### Consequences for forest management

This study addressed several of the ten highest-ranked questions regarding mixed-forest functioning and management, which Coll *et al.* (2018) identified by interviewing 168 managers from European countries. We considered which species combinations may be most beneficial (question 4), how is the productivity of mixtures different compared with that of mono-specific stands (question 5), which positive and negative effects mixtures can have and what is the balance or trade-off (questions 6 and 7); and finally, we reveal whether mixed stands are only denser or more efficient in resource use (question 10) (Coll *et al.*, 2018). Our results represent the mixing effects for medium- and high-quality site conditions. The range of site conditions of the used age series was not wide enough for exploring the dependency of the mixing effects on site quality. For the revelation of such dependencies transects of mono-specific and mixed-species stands along productivity gradients across Europe are more suitable (del Rio *et al.*, 2014; Heym *et al.*, 2017).

We found that mixing can be beneficial for trees and stands throughout the entire rotation. The finding that mixing increases leaf area efficiency means that the benefit of the mixture can also be exploited under lower stand densities. Any changes in maximum stand density are relevant for silvicultural stand assessment and density regulation. The natural maximum stand density is commonly used as a reference for defining density reductions in silvicultural guidelines. In this case, the site-specific maximum density was used as the ceiling density, and desired density trajectories were formulated in relation to the maximum. Density may increase the size growth and stability of remaining trees, but should be carefully assessed and regulated, as density reduction can also cause a decrease in stand growth. Thus, any neglect or underestimation of the maximum stand density may cause growth losses.

### CONCLUSIONS

The strong beneficial effects of species mixture and stand structure on tree and stand growth suggest potential research for the future. This study provides various new starting points for better understanding, design, and silvicultural steering, and exploits the benefits of mixed compared with mono-specific stands. The essential 3-D structure may be better and less costly, as measured by T-LiDAR in existing and newly established experiments. The species-specific behaviour suggests the avoidance of premature species-overarching generalization. The differentiation between density and efficiency effects offers promising starting points for further causal analyses and modelling mixing effects depending on site conditions. The attenuated trade-off between stand productivity and stem size growth enables increased stem diameter growth, even with similar or higher stand productivity and density compared with non-specific stands. The benefits of the mixture come on top of the other well-known superiority of provisioning and regulation services.

## SUPPLEMENTARY DATA

Supplementary data are available online at https://academic.oup.com/aob and consist of the following. Figure S1: visualizes by the example of Schongau 814 the setup of an age series by the crown maps of its corresponding plots SON 814/1–8 in 2011. Figure S2: demonstrates the method of plot edge correction by toroidal shift shown by example for SON 814/2 at the survey in autumn 2011. Figure S3: trade-off between stand mass growth and stem diameter increment in mixed compared with mono-specific stands for the mixtures Norway spruce/silver fir/European beech, Scots pine/Norway spruce and Scots pine/European beech. Tables S1–S4: parameters of the auxiliary relationships introduced in the Materials and methods section. Supplementary Derivation A: step-by-step derivation of eqn (11) introduced in the Statistical models section .

mcab077_suppl_Supplementary_Material_S01

mcab077_suppl_Supplementary_Material_S02

mcab077_suppl_Supplementary_Material_S03
