# Supplementary material for: Tree species mixing can increase stand productivity, density and growth efficiency and attenuate the trade-off between density and growth throughout the whole rotation
Source: Ann Bot. 2021 Jun 22;128(6):767–86. doi: 10.1093/aob/mcab077 (PMC8557385; doi:10.1093/aob/mcab077)
Supplement: mcab077_suppl_Supplementary_Material_S01 [file mcab077_suppl_supplementary_material_s01.docx]

Supplementary Tables 1 to 4

Supplementary Table 1 Species-specific models for the estimation of tree height in dependence on stem diameter and stand age The model functions are (1) $\ln\left( h \right)=a_{0}+a_{1}\times\ln\left( d \right)+a_{2}\times\ln\left( age \right)+a_{3}\times\ln\left( d \right)\times\ln\left( age \right)$ and (2) $\ln\left( h \right)=a_{0}+a_{1}\times\ln\left( d \right)$. All regression coefficients and models were significant, at least at the level of p<0.05.

| age series | species | Mo-del | n | a_0_ | a_1_ | a_2_ | a_3_ |
| --- | --- | --- | --- | --- | --- | --- | --- |
| NOR 811 | N. spruce | 1 | 551 | -4.15965 | 1.84940 | 1.35497 | -0.32143 |
| NOR 811 | E. beech | 1 | 899 | -0.58342 | 0.65887 | 0.56973 | -0.06076 |
| FRE 813 | N. spruce | 1 | 1406 | -4.56005 | 1.97956 | 1.47252 | -0.35217 |
| FRE 813 | E. beech | 1 | 1007 | 0.874962 | 0.557134 | 0.148499 | -0.009755 |
| SON 814 | N. spruce | 1 | 2439 | -3.1562814 | 1.4740666 | 1.1682324 | -0.2448174 |
| SON 814 | E. beech | 1 | 1749 | -0.8640346 | 0.9389290 | 0.5929986 | -0.1074265 |
|  |  |  |  |  |  |  |  |
| KRE 824 | N. spruce | 2 | 242 | -0.15555 | 0.88265 |  |  |
| KRE 824 | s. fir | 2 | 98 | 0.24168 | 0.77041 |  |  |
| KRE 824 | E. beech | 2 | 186 | 0.54871 | 0.66676 |  |  |
|  |  |  |  |  |  |  |  |
| GEI 832 | S. pine | 1 | 1092 | 0.848701 | 0.718827 | 0.004868 | -0.011401 |
| GEI 832 | E. beech | 1 | 744 | 1.05461 | 0.47314 | 0.08191 | 0.01347 |
| AMB 833 | S. pine | 1 | 1333 | -0.06474 | 0.82342 | 0.27796 | -0.04406 |
| AMB 833 | E. beech | 1 | 609 | 0.52614 | 0.50495 | 0.30772 | -0.01667 |
|  |  |  |  |  |  |  |  |
| NEU 841 | S. pine | 1 | 1307 | -0.252815 | 0.709393 | 0.641887 | -0.110573 |
| NEU 841 | N. spruce | 1 | 2685 | 1.64401 | 1.67509 | 0.55309 | -0.20694 |
|  |  |  |  |  |  |  |  |
| ROT 801 | s. oak | 1 | 621 | -2.593221 | 1.518920 | 1.063280 | -0.268847 |
| ROT 801 | E. beech | 1 | 649 | 0.19274 | 0.92671 | 0.24853 | -0.08147 |
| SWE 803 | s. oak | 1 | 736 | -1.256314 | 1.055488 | 0.782264 | -0.170763 |
| SWE 803 | E. beech | 1 | 741 | 1.26641 | 0.38526 | -0.06964 | 0.06479 |
| KEH804 | s. oak | 1 | 1366 | -0.057719 | 0.681863 | 0.510510 | -0.080530 |
| KEH804 | E. beech | 1 | 1364 | 1.19464 | 0.55845 | -0.04108 | 0.02801 |
|  |  |  |  |  |  |  |  |
| ARN 851 | E. ash | 1 | 876 | 1.18206 | 0.67263 | 0.01046 | -0.01021 |
| ARN 851 | s. maple | 1 | 1281 | 2.24106 | 0.29158 | -0.25633 | 0.07874 |

Supplementary Table 2 For estimation of the aboveground stem biomass depending on stem diameter, we used the species-specific functions by Forrester *et al.* (2017). The underlying model function is $\ln\left( ma \right)=a_{0}+a_{1}\times\ln\left( d \right)$. Factors CF were applied to correct for the bias that results from back-transforming ln-transformed predictions of the biomass, *ma* (Snowdon 1991). Stem mass was calculated according to $ma={exp(a}_{0}+a_{1}\times\ln\left( d \right))\times{CF}_{m}$.

For species with minor portions (e.g. *Taxus* spec., *Sorbus* spec.) for which species-specific functions were not available, we used the generalised functions for conifer and broad-leaved species, respectively (see also Forrester *et al.*, 2017).

| species | a_0_ | a_1_ | ${CF}_{m}$ |
| --- | --- | --- | --- |
| Norway spruce | -2.5027 | 2.3404 | 1.0599 |
| Scots pine | -2.3583 | 2.3080 | 1.0334 |
| silver fir | -3.2683 | 2.5768 | 0.9873 |
| European larch | -2.4105 | 2.4240 | 1.0186 |
| Douglas-fir | -2.9258 | 2.5980 | 0.9850 |
| sessile oak | -2.2131 | 2.3031 | 0.9724 |
| European beech | -1.4487 | 2.1661 | 0.9979 |
| European ash | -2.861 | 2.6921 | 0.9169 |
| sycomore maple | -2.5624 | 2.4634 | 0.9721 |
| conifers | -2.6216 | 2.4196 | 1.0573 |
| broad-leaved | -2.4521 | 2.4115 | 0.9362 |

Forrester DI, Tachauer IHH, Annighoefer P, et al. 2017. Generalized biomass and leaf area allometric equations for European tree species incorporating stand structure, tree age, and climate. *Forest Ecology and Management* 396: 160-175.

Snowdon P. 1991. Ratio estimator for bias correction in the logarithmic regressions. *Canadian Journal of Forest Research* 21(5): 720-724.

Supplement Table 3 For estimation of the leaf area per tree depending on stem diameter we used the species-specific functions by Forrester et al. (2017). The underlying model function was $\ln\left( la \right)=a_{0}+a_{1}\times\ln\left( d \right)$. Factors CF were applied in order to correct for the bias that results from back transforming ln-transformed predictions of the leaf area, la (Snowdon 1991). Leaf area was calculated according to $la={exp(a}_{0}+a_{1}\times\ln\left( d \right))\times{CF}_{la}$.

For species with minor portions (e.g. *Taxus* spec., *Sorbus* spec.) for which species-specific functions were not available, we used the generalised functions for conifer and broad-leaved species, respectively (see also Forrester *et al.*, 2017).

| species | a_0_ | a_1_ | ${CF}_{la}$ |
| --- | --- | --- | --- |
| Norway spruce | -1.3434 | 1.857 | 1.01109 |
| Scots pine | -2.065 | 1.746 | 1.00961 |
| European larch | -1.3776 | 1.7507 | 0.95729 |
| Douglas-fir | -1.6095 | 1.9025 | 0.97045 |
| sessile oak | -1.7839 | 2.1375 | 0.9663266 |
| European beech | -1.419 | 1.8909 | 1.09759 |
| European ash | -1.9073 | 2.3915 | 0.7927 |
| conifers | -1.8155 | 1.8971 | 0.984659 |
| broad-leaved species | -1.5623 | 1.8628 | 1.06626 |

Forrester, D. I., Tachauer, I. H. H., Annighoefer, P., Barbeito, I., Pretzsch, H., Ruiz-Peinado, R., ... & Sileshi, G. W. (2017). Generalized biomass and leaf area allometric equations for European tree species incorporating stand structure, tree age and climate. Forest Ecology and Management, 396, 160-175.

Snowdon, P. (1991). A ratio estimator for bias correction in logarithmic regressions. *Canadian Journal of Forest Research*, *21*(5), 720-724.

Supplementary Table 4 Mean equivalence factors for converting SDI from one species to another. The matrix shows the equivalence factors $e_{1\Rightarrow2}=SDIMAX_{2}/SDIMAX_{1}$for transforming the species-specific density of species 1 (entry via the left column) to species 2 (read off via the upper line). For example, the density of SDI= 900 in a Norway spruce stand is equivalent to a density of SDI = 567 in a European beech stand ($SDI_{E.be.}=SDI_{N.sp.}\times e_{1\Rightarrow2}$) with $e_{1\Rightarrow2}=0.63$ (according to Pretzsch and Biber, 2016; Fig. 4).

|  | Norway spruce | silver fir | Scots pine | European larch | European beech | sessile oak | Douglas- fir | black alder |
| --- | --- | --- | --- | --- | --- | --- | --- | --- |
| Norway spruce | 1.00 | 0.78 | 0.85 | 1.21 | 0.63 | 0.44 | 1.13 | 0.99 |
| silver fir | 1.29 | 1.00 | 1.09 | 1.56 | 0.81 | 0.56 | 1.45 | 1.27 |
| Scots pine | 1.18 | 0.92 | 1.00 | 1.43 | 0.74 | 0.51 | 1.33 | 1.16 |
| European larch | 0.83 | 0.64 | 0.70 | 1.00 | 0.52 | 0.36 | 0.93 | 0.82 |
| European beech | 1.60 | 1.24 | 1.35 | 1.93 | 1.00 | 0.70 | 1.80 | 1.58 |
| sessile oak | 2.29 | 1.78 | 1.94 | 2.77 | 1.43 | 1.00 | 2.58 | 2.26 |
| Douglas-fir | 0.89 | 0.69 | 0.75 | 1.07 | 0.56 | 0.39 | 1.00 | 0.88 |
| black alder | 1.01 | 0.79 | 0.86 | 1.23 | 0.63 | 0.44 | 1.14 | 1.00 |

Pretzsch H, Biber P. 2016. The mixing of tree species can increase the maximum stand density. *Canadian Journal of Forest Research* 46(10): 1179-1193.
