# Supplementary material for: Tree species mixing can increase stand productivity, density and growth efficiency and attenuate the trade-off between density and growth throughout the whole rotation
Source: Ann Bot. 2021 Jun 22;128(6):767–86. doi: 10.1093/aob/mcab077 (PMC8557385; doi:10.1093/aob/mcab077)
Supplement: mcab077_suppl_Supplementary_Material_S02 [file mcab077_suppl_supplementary_material_s02.docx]

Supplementary Derivation A

To analyse whether the tradeoff between stem size growth and stand productivity is modified by tree species mixing, we first fitted the relationships between stem diameter growth and stand density for mono- and mixed-species stands, $id=f_{1}(SDIc, m)$, and in the same way the relationships between stand growth and stand density for mono- and mixed-species stands.

$IMc=f_{2}(SDIc, m)$. This resulted in two equations:

$\ln\left( IMc \right)=a_{1}+b_{1}\times\ln\left( SDIc \right)+c_{1}\times m$

and

$\ln\left( id \right)=a_{2}+b_{2}\times\ln\left( SDIc \right)+c_{2}\times m$

Both equations were equalized, rearranged, and solved so that IMc was on the left and id on the right side $IMc=f_{3}(id, m)$.

$(\ln\left( IMc \right)-a_{1}-c_{1}\times m)/b_{1}=ln(SDIc)$

$(\ln\left( id \right)-a_{2}-c_{2}\times m)/b_{2}=ln(SDIc)$

$\ln\left( IMc \right)-a_{1}-c_{1}\times m=((\ln\left( id \right)-a_{2}-c_{2}\times m)/b_{2})\times b_{1}$,

$\ln\left( IMc \right)=b_{1}/b_{2}\times(\ln\left( id-a_{2} \right)+a_{1}-(c_{2}\times b_{1}/b_{2}-c_{1})\times m$

$IMc=e^{b_{1}/b_{2}\times(\ln\left( id-a_{2} \right)+a_{1}-(c_{2}\times b_{1}/b_{2}-c_{1})\times m}$

By inserting *m*=0 and *m*=1, respectively, this resulted in productivity-stem growth relationships for mono-specific and mixed species stands (see Fig. 8 in the main text).

It reflects the cost of stand productivity to increase the mean tree diameter by stand density reduction in monospecific stands, and how the relationship changes in mixed stands. As a simple measure, we introduce the ratio $r={IMc}_{mix}({id}_{2mm})/{IMass}_{mono}({id}_{2mm})$. For example, a ratio of $r=1.20$ indicates that the same diameter increment of 2 mm can be achieved with a 20 % higher productivity in mixed stands compared with monospecific stands because of the higher packing density and efficiency of the latter.
