# Supplementary material for: Tree species mixing can increase stand productivity, density and growth efficiency and attenuate the trade-off between density and growth throughout the whole rotation
Source: Ann Bot. 2021 Jun 22;128(6):767–86. doi: 10.1093/aob/mcab077 (PMC8557385; doi:10.1093/aob/mcab077)
Supplement: mcab077_suppl_Supplementary_Material_S03 [file mcab077_suppl_supplementary_material_s03.docx]

Supplementary Figures 1 - 3


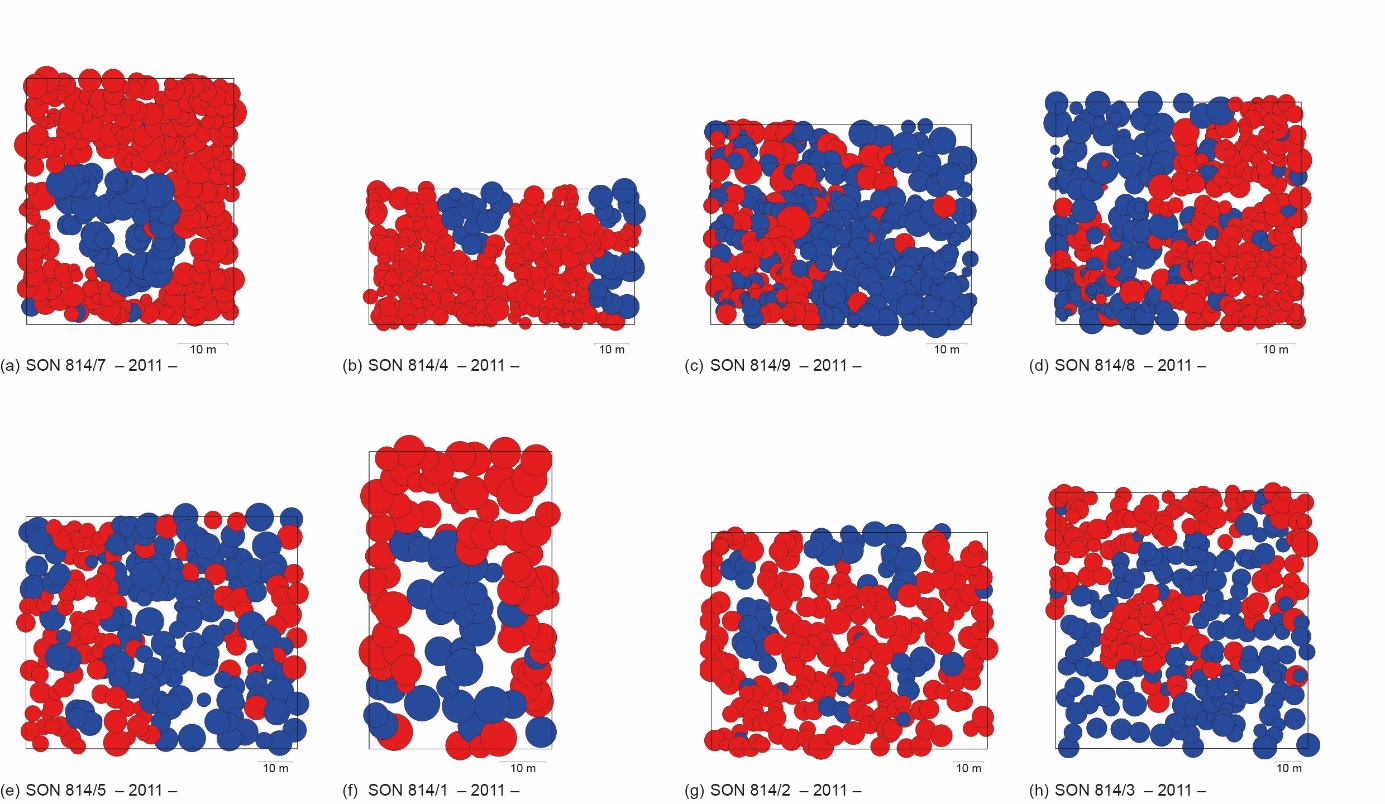


Supplementary Fig. 1 Example of one of the 11 age series used in this study. Setup of the age series SON 814/1–8 was visualised by the crown map in 2011. Eight plots (a-h) of the age series cover individual tree- and group-mixture Norway spruce (red) and European beech (blue) as well as mono-specific parts. The plot size increases from the young to the old stands (see 10 m scale at the bottom of each of the crown maps) in order to cover representative sections of the representative phases. For the sake of simplicity, we visualised the crown size by concentric circles calculated as the quadratic mean of the eight crown radius measurements recorded during the course of the repeated surveys. A pseudo 3D visualisation of the age series SON 814 is shown in Fig. 1 in the main text.

Supplementary Fig. 2 Plot edge correction by toroidal shift shown by example for SON 814/2 at the survey in autumn 2011. The plot 0 in the centre represents the original experimental plot with the standpoints of Norway spruces (red) and European beeches (blue). Before calculating the local SDI values and mixing proportions for neighbourhood analysis, we established a toroidal shift of the original plot 0 to all eight directions (duplicated plots 1, …8) of the plot periphery for edge bias compensation (Radtke and Burkhart, 1998; Pommerening and Stoyan, 2006; Pretzsch, 2009). Using the toroidal shift, we extended the same mixing pattern and distances in all eight directions and avoided any overestimation of density, as it could result from other techniques (Radtke and Burkhart, 1998).


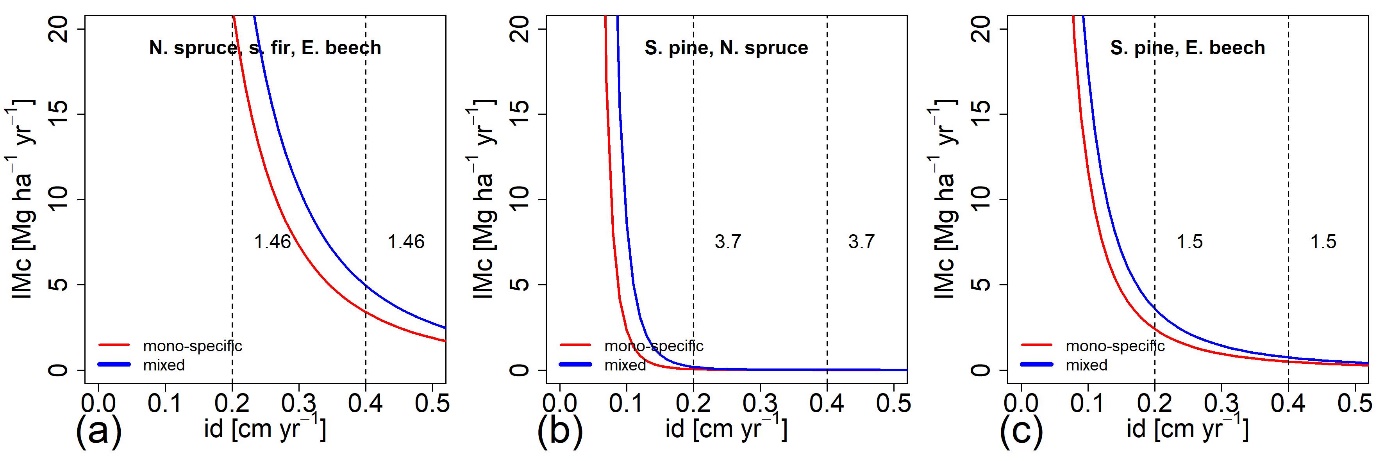


Supplementary Fig. 3 a-c Tradeoff between stand mass growth and stem diameter increment in mixed compared with monospecific stands, shown by example for the mixture of (a) Norway spruce, silver fir, European beech, (b) Scots pine and Norway spruce, and (c) Scots pine and European beech. For analogous relationships for the other three species mixtures, see Fig. 8 in the main text.
